# Target engagement and drug residence time can be observed in living cells with BRET

**DOI:** 10.1038/ncomms10091

**Published:** 2015-12-03

**Authors:** Matthew B. Robers, Melanie L. Dart, Carolyn C. Woodroofe, Chad A. Zimprich, Thomas A. Kirkland, Thomas Machleidt, Kevin R. Kupcho, Sergiy Levin, James R. Hartnett, Kristopher Zimmerman, Andrew L. Niles, Rachel Friedman Ohana, Danette L. Daniels, Michael Slater, Monika G. Wood, Mei Cong, Yi-Qiang Cheng, Keith V. Wood

**Affiliations:** 1 Promega Corporation, 2800 Woods Hollow Road, Fitchburg, Wisconsin 53711, USA; 2 Promega Biosciences Incorporated, 277 Granada Drive, San Luis Obispo, California 93401, USA; 3 UNT System College of Pharmacy, Department of Pharmaceutical Sciences, University of North Texas Health Science Center, Fort Worth, Texas, USA

## Abstract

The therapeutic action of drugs is predicated on their physical engagement with cellular targets. Here we describe a broadly applicable method using bioluminescence resonance energy transfer (BRET) to reveal the binding characteristics of a drug with selected targets within intact cells. Cell-permeable fluorescent tracers are used in a competitive binding format to quantify drug engagement with the target proteins fused to Nanoluc luciferase. The approach enabled us to profile isozyme-specific engagement and binding kinetics for a panel of histone deacetylase (HDAC) inhibitors. Our analysis was directed particularly to the clinically approved prodrug FK228 (Istodax/Romidepsin) because of its unique and largely unexplained mechanism of sustained intracellular action. Analysis of the binding kinetics by BRET revealed remarkably long intracellular residence times for FK228 at HDAC1, explaining the protracted intracellular behaviour of this prodrug. Our results demonstrate a novel application of BRET for assessing target engagement within the complex milieu of the intracellular environment.

## Abstract (web summary)

Drug molecules operate through physical interaction with specific cellular targets, and understanding this interaction is important for mechanisms and the potential therapeutic effect of drug candidates. Here, the authors show that bioluminescence resonance energy transfer can be used to monitor the intracellular engagement of a drug with its target.

## Graphical abstract

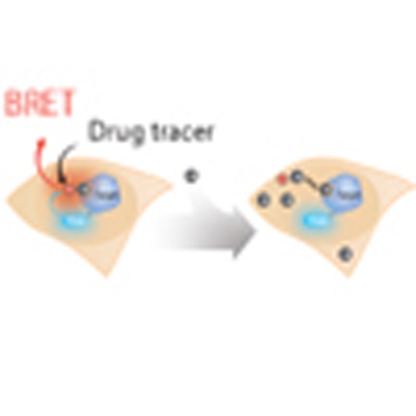

Deciphering how small molecule modulators bind their intracellular targets is fundamental to understanding pharmacological mechanism. In addition to the specificity and affinity of target engagement, binding dynamics under non-equilibrium conditions may also underlie the therapeutic potential of new drug candidates^1^,^2^,^3^. These parameters are routinely assessed through biochemical means, which may fail to adequately mimic the complexity of the intracellular environment. Proteins reside in structurally intricate settings within the cells and typically function as components of extended molecular complexes, and thus they may exhibit significantly different behaviours than they would as isolated polypeptides^4^,^5^,^6^,^7^. It is not surprising that biochemical analysis of target engagement often fails to correlate with compound potency measured by cellular phenotype. Preferably, correlations between binding interactions and physiological outcomes should be made within a common physiological context. For this reason, the pharmaceutical industry has directed increased efforts towards assessing target engagement within intact cells^8^,^9^,^10^.

While quantitation of compound binding to purified proteins or surface receptors (in particular G-protein coupled receptors) is well established^11^,^12^,^13^, similar analysis for intracellular targets has been more difficult. Indirect approaches are often used instead, relying on deconvolution of cellular responses to infer target engagement^14^. For example, expression profiling may be used as an indicator of altered target activity in response to agonists or antagonists. However, compounds typically bind to multiple targets within cells, where only a few are mechanistically associated with the relevant phenotype. Unambiguously resolving the molecular targets of compounds within complex pathways and establishing that a cellular response serves as an adequate proxy for physical binding by the compound can be challenging.

More recently, various qualitative approaches based on ligand-induced protein stabilization have been used to characterize target engagement^9^,^10^,^15^,^16^. Such methods can be limited by the incremental stability imparted by compound binding relative to the inherent stability of the intracellular target. Consequently, these methods are prone to false negative results as many targets fail to exhibit measurable stabilization upon ligand binding^17^. For some of these techniques, elevated temperatures are required for the analysis, and thus may not represent physiological conditions for compound binding. Importantly, these methods are limited to end point analysis, complicating the application of such methods for measurements of binding kinetics or compound residence time.

Assessments of target engagement are especially challenging for prodrug inhibitors that require intracellular activation for maximal potency^18^,^19^,^20^. Mechanistic studies for such prodrug inhibitors may not be adequately represented in a biochemical framework, and may require analysis in cells to be physiologically meaningful. For example, the clinically approved histone deacetylase (HDAC) prodrug FK228 (depsipeptide, romidepsin, Istodax) as well as the related natural product thailandepsin A (TDP-A) utilize a unique mechanism that require intracellular reduction to achieve maximal potency^18^,^19^,^21^. It has been recently demonstrated that pulse-treatment of cells with FK228 results in highly potent and persistent inhibition of pan-HDAC activity^22^,^23^,^24^. Although various alternate intracellular mechanisms have been proposed for this observation^24^, it has not been determined whether the sustained potency of FK228 is mechanistically associated with the intracellular residence time at HDAC isozymes. Biophysical methods compatible with living cells are therefore needed to interrogate target engagement and residence time for this compound class.

Bioluminescence resonance energy transfer (BRET) can reveal real-time molecular interactions within intact cells without cell lysis or non-physiological temperatures^25^. Energy transfer techniques such as BRET or fluorescence resonance energy transfer (FRET) are well established for quantifying intracellular protein–protein interactions within cells; however, BRET is often preferred owing to increased detection sensitivity^26^,^27^,^28^. While both energy transfer techniques have been utilized to measure compound binding to extracellular or lysate-derived analytes^12^,^13^,^29^,^30^, neither has been successfully applied to the interrogation of target engagement and compound residence time within intact cells.

In contrast to previous applications of energy transfer, the approach presented here utilizes live cells expressing an intracellular target protein genetically fused to NanoLuc luciferase and a cell-permeable fluorescent tracer derived from a suitable drug or tool compound. BRET is achieved inside intact cells by reversible binding of the fluorescent tracer to the intracellular target. The binding characteristics of an interacting compound are revealed by its ability to compete with the tracer and thus influence the production of BRET. The general applicability of this approach for intracellular proteins is supported by our ability to quantify intracellular engagement by inhibitors of HDACs, bromodomains (BRDs) and kinases. Our analysis focused on target engagement at HDACs because this new capability should enable the biophysical characterization of target engagement and residence time for a diverse set of HDAC inhibitors, including the prodrugs FK228 and TDP-A. The BRET approach enabled a mechanistic interrogation on the enhanced potency and sustained efficacy of FK228, allowing us to directly measure both target engagement and residence time against target HDACs within intact cells. Using BRET, we were able to determine that the prolonged phenotypic effects of FK228 and TDP-A prodrugs in non-equilibrium conditions are due to the remarkably stable complexes formed between these molecules and HDAC protein within cells.

## Results

### BRET enables target engagement analysis for HDACs

**Figure 1:**
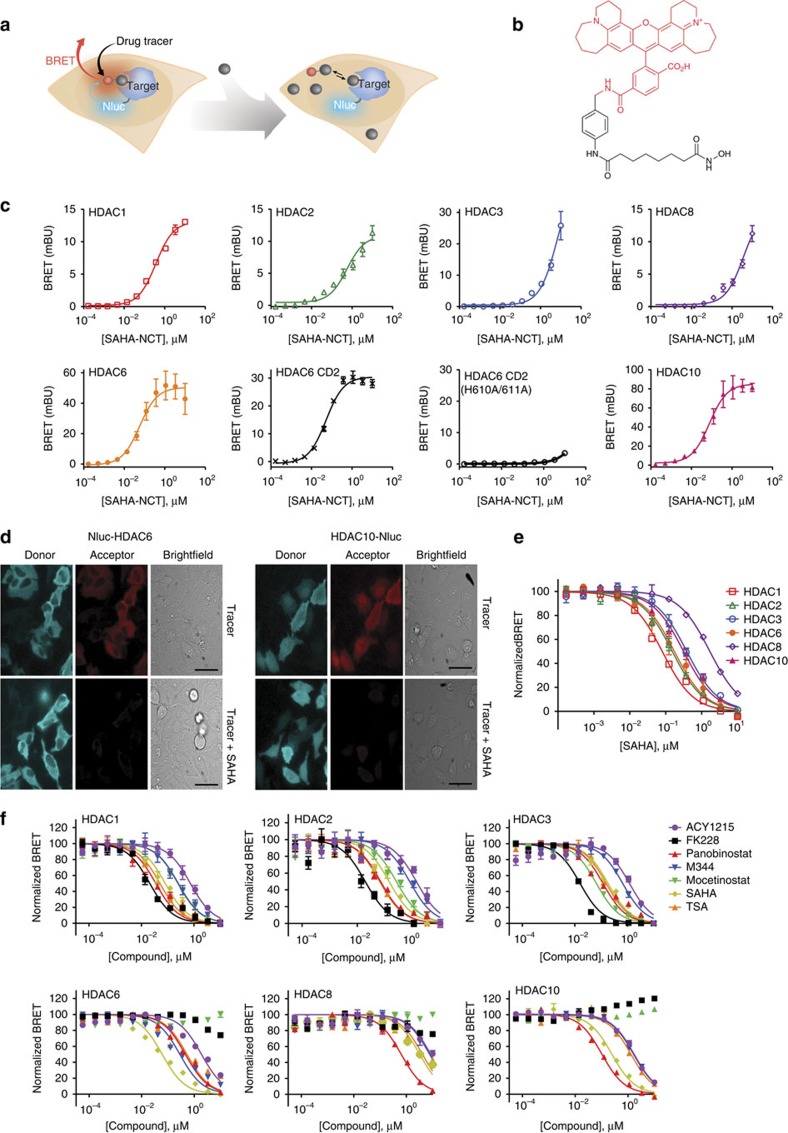
Measuring target engagement at intracellular HDACs. (**a**) Illustration of intracellular target engagement assay. A permeable fluorescent tracer binds in dynamic equilibrium to an intracellular target protein fused to Nluc, resulting in BRET. Introduction of compounds that bind the same target cause the tracer to be displaced, resulting in a decrease in BRET. (**b**) The HDAC tracer was derived from the adduct of a broad-coverage HDAC inhibitor (SAHA, shown in black) and the NCT dye (shown in red). (**c**) Introduction of SAHA-NCT to cell medium resulted in specific and concentration-dependent intracellular BRET with Nluc fusion of various HDACs (classes I and IIb) expressed in HeLa cells, as measured using a microplate luminometer. A control HDAC6 CD2 construct encoding a binding-deficient mutant (H610A/H611A) showed weakened engagement with the tracer. BRET values at each tracer concentration were background-corrected by parallel measurements made in the presence of an excess of unmodified SAHA (20 μM) (as described in Methods and Supplementary Fig. 1a). Apparent tracer affinities were estimated using equation (1) and reported in Supplementary Table 1. Data are mean±s.e.m. of three independent experiments. (**d**) Luminescence images of HeLa cells expressing HDAC6 or HDAC10 fused to Nluc, shown with false colouring to represent light emission from 420–500 nm (donor) or above 590 nm (acceptor). Images were acquired in the presence of SAHA-NCT tracer alone (1 μM), or in combination with excess unlabelled SAHA (10 μM). Signal suppression caused by excess SAHA indicates specificity of the BRET signal. (**e**) Concentration-dependent attenuation of BRET from intracellular HDAC fusions with titration of SAHA in the presence of 1 μM SAHA-NCT tracer. Data were collected on a microplate luminometer and are mean±s.e.m. of three independent experiments. See Supplementary Table 1 and equations (2) and (3) for estimation of apparent affinities of SAHA to HDACs. (**f**) BRET measurements showing the relative affinity of HDAC inhibitors in HeLa cells (1 μM SAHA-NCT). See Supplementary Table 2 for relative compound affinities to individual HDACs. Data are mean±s.d. of four data points.

We used a small luciferase (NanoLuc/Nluc, 19 kDa) as a BRET donor, which has been recently demonstrated to produce extraordinarily intense and stable luminescence with a relatively narrow spectral distribution^31^. For BRET tracers, we applied Non-Chloro-TOM dye (NCT) as a BRET acceptor, which demonstrated adequate cell-permeability and provided significant spectral resolution from Nluc^25^. Detection of tracer binding by energy transfer should be broadly applicable to intracellular proteins so long as the tracer is permeable to the cells and the proteins can be suitably tagged with Nluc. Intracellular target engagement is measured by competitive displacement of the tracer resulting in a loss of BRET (Fig. 1a).

HDACs served as the primary model system, where a single broad-coverage tracer could enable analysis of target engagement over multiple classes of HDACs. To elucidate inhibitor interactions across classes I and IIb HDACs, a broad-coverage target engagement assay was developed using a fluorescent tracer derived from SAHA coupled to NCT dye (Fig. 1b). Intracellular binding of the SAHA-NCT tracer to selected HDAC isozymes was detected by measuring the bioluminescence emission from a population of cells expressing a genetic fusion between the isozyme and Nluc. The putative targets of SAHA^32^; classes I and IIb HDACs, were included in the study to reveal isozyme-specific differences in inhibitor affinity. As HDACs coordinate binding of hydroxamic acid-based inhibitors such as SAHA via two highly conserved active-site His residues^33^,^34^, a binding-deficient construct encoding Nluc-HDAC6 CD2 (H610A/H611A) was included in the analysis as a negative control. Furthermore, class IIa HDACs (HDACs 4, 5, 7 and 9) and class IV HDAC (HDAC11) were also included as controls because of their expectedly low affinity to the SAHA-NCT tracer^32^. Microplate measurements of HeLa cells transiently expressing the genetic fusions confirmed both specific and concentration-dependent BRET only for classes I and IIb HDACs (Fig. 1c, Supplementary Fig. 1). To determine specific BRET signal, BRET data were background-corrected using a molar excess of unlabelled SAHA (Supplementary Fig. 1a). Both N- and C- terminal fusions were evaluated, with the orientation producing the largest BRET ratio for each isozyme shown in Fig. 1c. HDACs are known to tolerate reporter tags or purification tags larger than Nluc^35^, and we found that tethering of Nluc did not significantly alter HDAC activity, using HDAC2 as a an example (Supplementary Fig. 1c).

The highest tracer affinity was evident for HDACs 1, 2, 6 and 10, with lower affinity observed for HDACs 3 and 8 (Fig. 1c). While the presence of the fluorescent moiety on SAHA impacted affinity of the tracer, the observed rank-order engagement by the SAHA-NCT tracer was in general agreement with the relative affinities reported for unmodified SAHA^32^ (Supplementary Table 1). Because of the high luminescence intensity of Nluc interaction of the SAHA-NCT tracer could be detected at expression levels comparable to the endogenous protein, as demonstrated for HDAC1 in multiple cell types (Supplementary Fig. 1d). These results support that HDAC-Nluc fusions do not require overexpression to detect target engagement analysis via BRET.

In contrast to other members of the class I/IIb HDAC family, the HDAC6 isotype possesses two putative catalytic domains (CDs) as determined by homology analysis^36^,^37^,^38^. To determine whether the SAHA-NCT tracer binds one of the domains preferentially, we generated Nluc fusions with segregated CDs of HDAC6 and queried tracer binding in cells. BRET measurements demonstrated engagement of the tracer with the C-terminal CD (CD2) (Fig. 1c), whereas negligible BRET signal was observed at CD1 (Supplementary Fig. 1b). It has been reported that CD1 may lack deacetylase activity, and our results suggest that altered binding specificity may contribute to this^38^. As expected, the HDAC6 CD2 (H610A/H611A) mutant engaged SAHA-NCT poorly as determined via BRET (Fig. 1c).

To verify that BRET was arising from within living cells rather than from cellular debris that may be present in the culture medium, we imaged the luminescence emission from individual cells expressing Nluc fusions of HDAC6 or HDAC10. The addition of the SAHA-NCT tracer (1 μM) resulted in observable BRET clearly located within HeLa cells transiently expressing Nluc fusions of HDAC6 or HDAC10 (Fig. 1d, Supplementary Fig. 1e). Challenge with a molar excess of unmodified SAHA (10 μM) resulted in attenuation of the BRET signal, indicating that the BRET signal was generated by a specific and reversible interaction of the tracer with the Nluc fusion protein. BRET signal was observed exclusively in the cytoplasm of HeLa cells expressing Nluc-HDAC6, whereas HDAC10-Nluc expressing cells generated BRET in both the nucleus and cytosol. This is consistent with the expected localization patterns of both HDAC6 and HDAC10, and further demonstrates that the BRET signal is imposed by the placement of the luciferase even though the SAHA-NCT tracer is expected to bind multiple intracellular HDACs^36^.

The ability to displace the tracer by an unlabelled compound allowed the relative binding efficiency by HDAC inhibitors to be determined for multiple intracellular isozymes. Unlabelled SAHA effectively attenuated the BRET signal in a concentration-dependent manner when added to cells in combination with the SAHA-NCT tracer (Fig. 1e). The relative binding efficiency of SAHA for different HDAC isozymes is apparent by the concentration required to displace a defined amount of the tracer. Using a fixed concentration (1 μM) of SAHA-NCT for competitive displacement, relative binding efficiencies were estimated by the Cheng-Prusoff equation^39^ (see Supplementary Table 1 for observed and adjusted values). By this method, the greatest binding was observed with Nluc fusions of HDAC1, 2, 6 and 10, whereas HDACs 3 and 8 showed lower relative binding, as expected.^32^ Although SAHA and the SAHA-NCT tracer share the same binding motif, this approach should be applicable to any molecule able to competitively displace the tracer from the target.

### Profiling engagement and potency of HDAC inhibitors in HeLa

To demonstrate the utility of the BRET method to profile intracellular target engagement across HDACs, we used a structurally diverse set of inhibitors that included both hydroxamate- and non-hydroxamate-based compounds. Hydroxamate-based inhibitors were represented by SAHA, trichostatin A (TSA), ACY1215, M344 and panobinostat. Mocetinostat is an aminobenzamide-based inhibitor, and FK228 is a bicyclic depsipeptide prodrug. All six of the tested inhibitors engaged HDACs 1, 2 and 3, with FK228 demonstrating the highest engagement for these isozymes (Fig. 1f). Negligible binding of FK228 and mocetinostat were observed for HDAC6, HDAC8 and HDAC10 (Fig. 1f). Rank-order binding of the compounds for HDACs 1 and 2 were in agreement, likely explained by the close sequence homology between these proteins^40^. Other HDAC isozymes had more varied selectivity profiles, providing selectivity signatures that were largely in agreement with previous reports, albeit with some exceptions^32^,^40^. The bicyclic depsipeptide inhibitors of HDACs (including FK228 and the recently described TDP-A) are notable in that they rely on a unique mechanism of prodrug activation which requires intracellular reduction of a disulfide bond^18^,^19^,^41^. The ability to observe target engagement within living cells thus allows characterization of inhibitor mechanism for these compounds within the physiological environment necessary for prodrug conversion.

**Figure 2:**
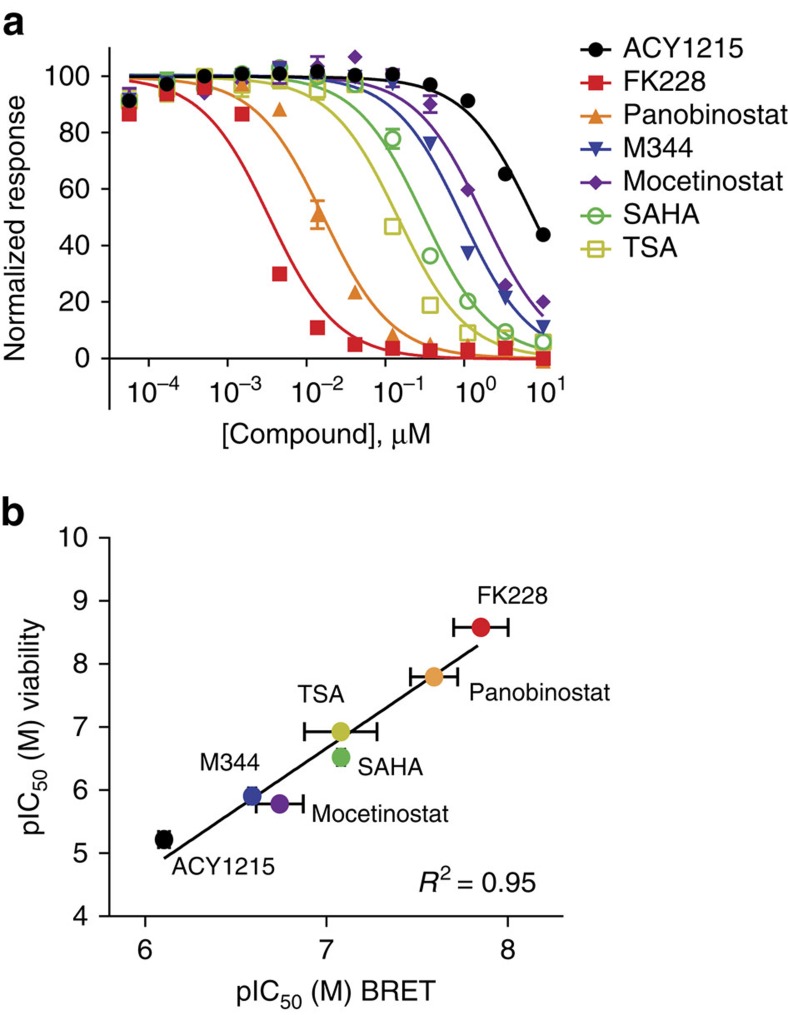
Correlation of phenotypic potency with target engagement to intracellular HDAC isozymes. (**a**) 48 h treatment with HDAC inhibitors results in antiproliferative effects in HeLa cells, as measured by intracellular ATP levels. Data are the mean of three independent experiments±s.e.m.. (**b**) Antiproliferative potency of HDAC inhibitors, as determined by cellular ATP levels in HeLa cells, is strongly correlated with target engagement to HDAC1. See Supplementary Fig. 2b and Supplementary Table 2 for the comparative correlation with other HDAC isozymes. Data are the mean of 3 independent experiments±s.d.

It is well established that upregulated HDAC 1 and 2 activities inhibit apoptosis and mediate proliferation in cervical cancer cell lineages such as HeLa^40^,^42^,^43^. To verify that our intracellular target engagement profiles measured by BRET accurately correlate with antiproliferation, we determined potencies for the panel of inhibitors using assays of apoptosis (caspase 3/7 activation) and cell proliferation (cellular ATP). A range of potencies was observed with similar rank-order produced by either method (Fig. 2a, Supplementary Fig. 2a). As expected^40^, target engagement was found to strongly correlate with antiproliferation for HDAC1 and HDAC2, yielding R^2^=0.95 and 0.88 respectively (Fig. 2b, Supplementary Fig. 2b and Supplementary Table 2). Unsurprisingly, the correlations with less relevant HDACs were relatively poor (Supplementary Fig. 2b, Supplementary Table 2). As HDAC1 and HDAC2 are the dominant HDACs mediating cell proliferation in many cancer cell lines, these correlations reinforce the validity of the target engagement measurements provided by BRET^40^,^42^,^43^.

### BRET reveals long residence times for FK228 and TDP-A

**Figure 3:**
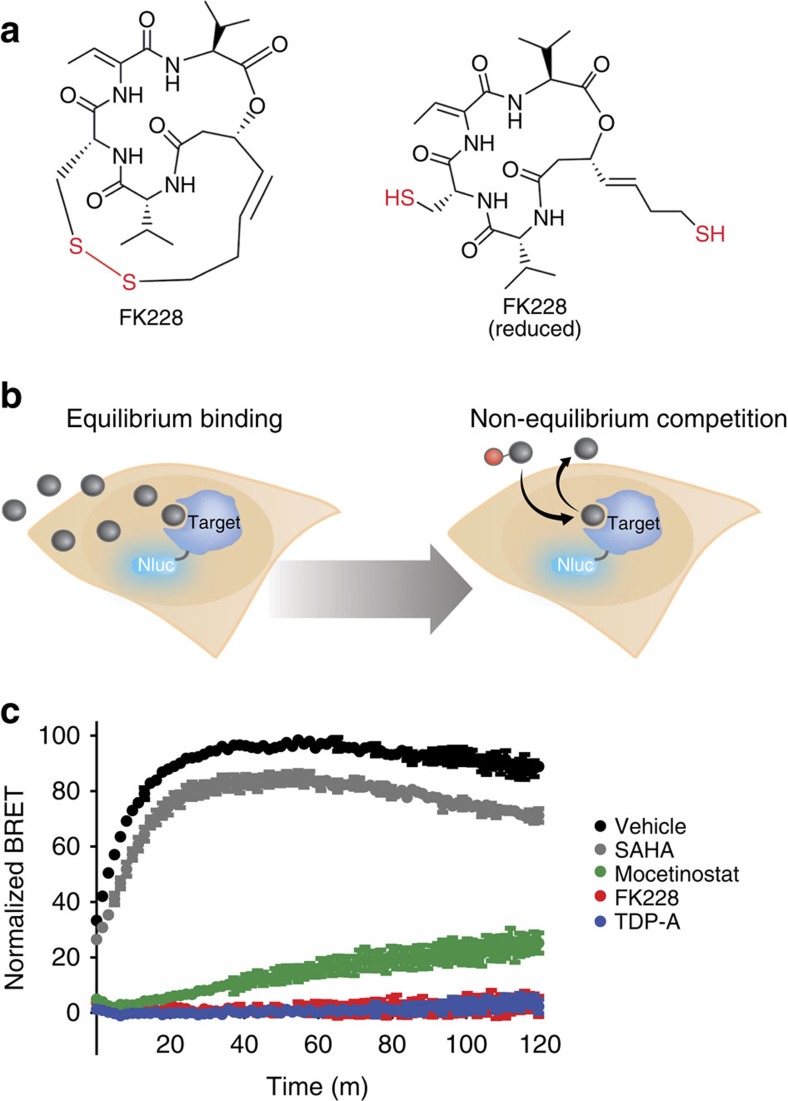
Measuring the intracellular residence time of HDAC inhibitors at HDAC1. (**a**) Structure of the inhibitor FK228 in the prodrug form (left) and the form activated by intracellular reduction (right). (**b**) Illustration of assay method for measuring intracellular residence time using BRET. Live cells expressing the target protein fused to Nluc are equilibrated with a near-saturating concentration of compound. The cells are then washed to remove unbound compound and treated with a near-saturating concentration of a tracer. The profile of compound with slow dissociation kinetics from the target impedes tracer binding, which slows production of the BRET signal. (**c**) Residence time analysis by BRET reveals remarkably slow dissociation rates for FK228 (red) and TDP-A (blue), compared with mocetinostat (green), SAHA (grey) or vehicle/DMSO control (black). Data are normalized to maximum signal (vehicle/DMSO-treated) versus full-occupancy control (10 μM SAHA, no washout). The kinetic traces for FK228 and TDP-A are nearly indistinguishable from the full occupancy control (10 μM SAHA, no washout). Data are mean±s.e.m.. of four independent experiments.

FK228 and related TDP-A require the reducing environment within cells to activate their sulfhydryl groups and achieve maximal potency^18^ (Fig. 3a). Although there has been speculation regarding the possible causes for protracted efficacy of FK228 in pulse-treatment experiments^22^,^24^, the precise mechanism has not been determined. We investigated this for FK228 and TDP-A by analysing temporal changes in the BRET measurements to characterize kinetic aspects of target engagement. As a prerequisite, it was necessary to establish that SAHA-NCT demonstrates rapid binding kinetics to ensure that dynamic interaction of the tracer with the intracellular target remains close to equilibrium over the time interval needed for engagement by the inhibitors^44^. As SAHA has fast binding kinetics, the SAHA-NCT tracer should be suitable for kinetic analysis of relatively slow-binding HDAC inhibitors in a competitive format^45^. Addition of a 1 μM solution of tracer to cells expressing HDAC1-Nluc resulted in a rapid increase in BRET, reaching a plateau within ∼20 min of tracer addition (Supplementary Fig. 3a). This rate is at least partially impacted by permeability across the plasma membrane, as addition of digitonin as a permeabilizing agent resulted in near-instantaneous BRET between the tracer and HDAC1-Nluc (Supplementary Fig. 3e).

Unlike hydroxamate-based inhibitors such as SAHA, it as been previously reported that prodrug inhibitors in the FK228 family possess a slow but protracted activity in cancer cell lineages^23^. Using the BRET approach, we confirmed that fast binding kinetics are observed for unmodified SAHA in HeLa cells (Supplementary Fig. 3b). In contrast to SAHA, FK228 and TDP-A equilibrated slowly, displaying an IC_50_ value that dropped continuously over the 180 min timecourse. The slow-binding kinetics of the prodrug inhibitors in cells were corroborated in an enzymatic assay using purified HDAC1 and chemically reduced FK228 or TDP-A (Supplementary Fig. 3c). Our results therefore support previous observations regarding the delayed effect of FK228 inside cancer cells.

Because of the reported functional persistence of FK228 activity, it has been suggested that these inhibitors may accumulate within cells, impact HDAC expression levels, or release slowly from their targets^22^,^24^. To directly assess the possibility of slow release from intracellular HDAC protein, the BRET method was reconfigured to measure the relative rates of compound dissociation from HDAC1. HeLa cells expressing HDAC1-Nluc were first equilibrated with a saturating concentration of compound. The aminobenzamide inhibitor mocetinostat was included as a control, as it has been demonstrated that inhibitors in this class possess slow dissociation kinetics with HDAC1 (refs 45, 46, 47). Cells were treated with FK228 at 100 nM, TDP-A at 10 nM, mocetinostat at 10 μM, or SAHA at 10 μM, followed by ligand removal (including an additional wash for FK228 and TDP-A) and immediate challenge with a saturating concentration of SAHA-NCT tracer. Increased BRET is indicative of tracer binding to the HDAC, which cannot occur until after the compound has dissociated (Fig. 3b, see Supplementary Fig. 3d for a detailed schematic representation of the assay). This analysis was also performed in lysed cells to address the proposed possibility that the reduced FK228 and TDP-A could accumulate and become trapped within cells, leading to residual target engagement after compound removal^22^,^24^. In live cells, dissociation of SAHA from HDAC1 was rapid and beyond the detection limits of the assay, whereas the reference compound mocetinostat displayed a slower dissociation rate as expected (Fig. 3c). The intracellular dissociation rate for FK228 and TDP-A were slower than the control compound mocetinostat at HDAC1, with a residence time profile similar to fully bound HDAC1 (samples without 10 μM SAHA removal, Fig. 3c). Results were corroborated in lysed cells, suggesting that intracellular trapping of FK228 is insufficient to explain the observed kinetics of dissociation (Supplementary Fig. 3e). These results indicate a remarkably slow dissociation rate (long residence time) for FK228 and TDP-A from HDAC1, and suggest that the strong binary complex between the inhibitors and HDAC protein are the cause of the prolonged phenotypic effect.

### BRET for target engagement at alternate target classes

**Figure 4:**
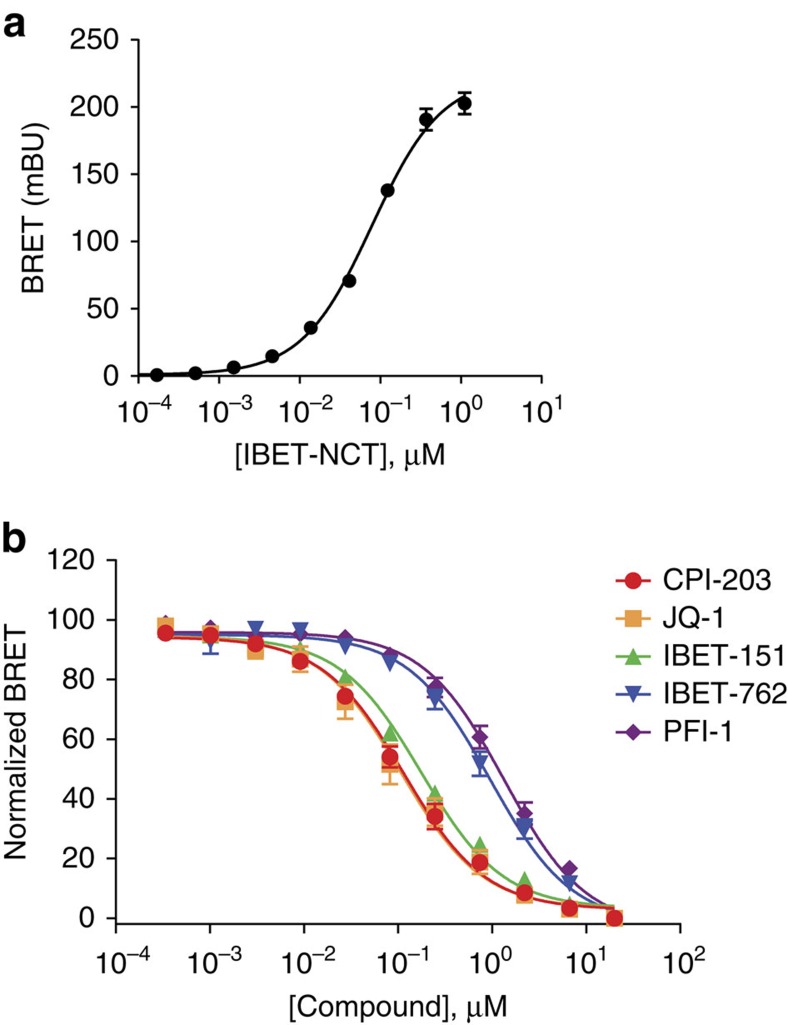
Measuring intracellular target engagement at bromodomain BRD4 using BRET. (**a**) Specific and dose-dependent BRET was observed with Nluc-fusions to full-length BRD4 expressed in HeLa. BRET values at each tracer concentration were background-corrected by parallel measurements made in the presence of an excess of unmodified compound (20 μM) (described in Methods). Data are mean±s.d. of four data points. (**b**) Use of BRET to measure rank-order affinity of BRD inhibitor panel against BRD4 within live HeLa cells (1 μM IBET-NCT held constant). Data are mean±s.e.m. of four independent experiments.

In addition to HDACs, we evaluated the BRET method for the BET family of BRDs and the lymphocyte specific protein tyrosine kinase (LCK) using BRET tracers derived from known inhibitors of these target proteins (Supplementary Fig. 4a, Supplementary Methods). An NCT tracer derived from iBET-762 engaged full-length Nluc-BRD4 proteins (Fig. 4a, Supplementary Figure 4b), as well as other members of the BET family of BRDs (Supplementary Fig. 4c). Target engagement profiles at BRD4 were then determined in competitive displacement formats with a panel of known BRD4 inhibitors (Fig. 4b) as well as segregated domains of BRD4 (Supplementary Fig. 4d). Applicability to intracellular kinases was then demonstrated at LCK using an NCT tracer derived from BIBF-1120 (Supplementary Fig. 4e,f). For both BRDs and LCK, rank-order affinities of various reference compounds observed using BRET were consistent with previous reports^48^,^49^,^50^ Taken together, these results indicate that energy transfer to cell-permeable tracer may provide a broadly applicable means for evaluating target engagement over key intracellular target classes.

## Discussion

This report describes the first method to directly assess target engagement and binding kinetics within intact mammalian cells under physiological conditions. Compound binding is detected by BRET, a biophysical process which provides significant advantages over alternative methods that rely on more complex biological proxies, operate under non-physiological or disruptive conditions, or are unsuitable for real-time analysis of compound binding and dissociation^10^,^15^. The simplified workflow we describe also eliminates the laborious liquid transfer steps and excessive hands-on time required of many alternative methodologies, particularly those utilizing thermal denaturation. Compared with biochemical methods, detection of intracellular target engagement provides an assessment of binding efficiency in an environment more representative of the cellular milieu where it would occur in tissues. The intracellular measurements also circumvent the need for isolated enzymes, which may be difficult to obtain by heterologous expression with sufficient purity and in a structurally appropriate form. This is the case with HDACs, for example, which generally function in protein complexes^51^.

The ratiometric nature of energy transfer measurements mitigates potential assay interferences, such as variations in cell number or expression level between samples^26^,^27^,^52^. BRET was chosen as the preferred energy transfer system over FRET due to the increased detection sensitivity it provides in microplate formats^26^,^27^,^28^,^30^,^52^,^53^. NanoLuc, in particular, provides excellent sensitivity as a BRET donor because of its small and stable structure, exceptionally bright luminescence, and relatively narrow emission spectrum^31^. Corroborating the signal strength and sensitivity of this method within live cells, energy transfer between Nluc and the NCT tracers produced sufficient BRET signal to directly image target engagement at the single-cell level using bioluminescent imaging microscopy. Using this BRET technique, it may be therefore possible to study target engagement distribution over a cell population.

The binding profiles for the tracers described in this report provide evidence that the red-emitting fluorophore, NCT, can be coupled onto structurally diverse chemical probes to provide cell-permeable BRET tracers for a variety of target classes. For tracer derivatization, we utilized probes with known mechanism of action and putative target engagement profiles. Probe molecules with less established structure–activity relationships may represent additional challenge for NCT modification. In the context of phenotypic screening (when knowledge of target selectivity for a lead compound is unknown), it may be useful to explore the suitability of the BRET technique for target identification. As an extension of the principle demonstrated here, it may be possible to apply an NCT derivative of a lead compound against a diverse panel of Nluc fusion constructs as a screen for potential intracellular targets. Such an approach could serve as a complement to more traditional mass spectrometry-based methods for target identification.

Although the fluorescent moiety of the tracer may impart altered target affinity compared with the unlabelled parent compound, our results support general applicability of the method so long as the tracer is permeable to cells, specific BRET can be measured over a titratable concentration range, and that competitive displacement can reveal accurate target engagement profiles for unlabelled compounds. However, use of BRET tracers for target engagement studies may represent a limitation for mechanistic studies on allosteric ligands that occupy binding sites distinct from that of the tracer. Ligands that engage the target but fail to compete with the tracer would therefore represent false negatives. While this may represent a potential liability, it has been well-documented that allosteric ligands may bind in a mutually exclusive manner with orthosteric (active-site) tracers^54^. Future studies are warranted to determine if this BRET technique could enable target engagement studies in this context. Non-tracer-based techniques (such as those utilizing thermal stability or thermal shift) are prone to a distinct set of false negatives, as high affinity ligands may not impart a measurable increase in stability for certain target proteins^17^. As this BRET technique is independent of target stability or denaturation steps, it may be a useful complement to stability-based assessments of target engagement.

By using a broad-spectrum tracer for analysis of class I/IIb HDACs, we successfully demonstrated that a single tracer could enable a target engagement study across multiple classes of HDACs. Although our engagement results were largely in agreement with literature^32^,^40^, small discrepancies could be attributed to the behaviour of isolated proteins versus the intracellular targets interrogated in our analysis. Indeed, there is a lack of consensus in the literature regarding the profiles of our compound set against purified HDACs^4^,^32^,^40^, possibly explained by the diversity and variability of available biochemical HDAC assay components.

To evaluate the validity of our HDAC target engagement results, we performed the first systematic correlation analysis between intracellular isozyme affinity and antiproliferative potency in a common cellular context. Target engagement measured by BRET for HDAC1 and HDAC2 for the compound panel showed a strong correlation with both apoptosis and proliferation of HeLa cells. The involvement of HDACs 1 and 2 in these phenotypic outcomes is consistent with proposed models for their roles in tumorigenesis in cervical cancer cells such as HeLa. This relationship is not surprising, as HDAC1 and HDAC2 share 87% sequence similarity and form intracellular protein complexes which coordinate the transition from G1 to S-phase in the cell cycle^42^,^43^. They are also commonly upregulated in tumour cells, including HeLa. Consequently, it is not surprising that pharmacological inhibition of these enzymes should inhibit proliferation in this cell model^40^. While a strong correlation was observed for HDACs 1 and 2 (and to a lesser extent HDAC3), the relatively poor correlation observed at HDACs 6, 8 and 10 support that these targets are not directly implicated in the antiproliferative effects of HDAC inhibitors in this cell model. Our observed profiles for target engagement therefore corroborate the proposed link between HDAC1 and HDAC2 activities in tumour cell proliferation and inhibition of apoptosis.

Although assessments of target occupancy are often assessed under equilibrium (using affinity values such as K_d,_ IC_50_), this may not be adequate for predicting target occupancy *in vivo*, where equilibrium conditions may not apply^1^. Under the dosing dynamics that are representative of *in vivo* conditions, dissociation rate, or residence time, may better characterize the ligand-receptor complex^1^,^2^,^3^. Furthermore, intracellular residence time may be impacted by intracellular target densitites, suggesting that binding analysis using purified analytes may not be adequate to predict drug residence time *in vivo*^7^. However, no methods currently exist to directly measure intracellular target engagement under non-equilibrium conditions. To enable a biophysical assessment of this process as it occurs inside intact cells, we explored the feasibility for using intracellular BRET to observe the kinetics of drug dissociation for a select group of HDAC inhibitors.

To mechanistically characterize the persistent effects of FK228 following inhibitor washout experiments, we configured the BRET assay to directly monitor dissociation of the prodrug inhibitors at HDAC1 inside cells. In this configuration, extremely slow apparent dissociation kinetics (long residence time) were observed for these HDAC inhibitors compared with SAHA. Moreover, the prodrug inhibitors showed even longer residence time than mocetinostat, a compound with reportedly slow dissociation rates at HDACs^45^,^46^,^47^.

Consistent with our residence time analysis, it has been well-documented that pulse-treatment of cells with FK228 results in enhanced and persistent inhibition of intracellular HDAC activity compared with SAHA^22^,^24^. This enhanced duration of HDAC inhibition was concomitant with increased HIV RNA expression and virion release from infected T-cells, indicating that FK228 should be further assessed for treatment of latent viral reservoir in HIV-infected patients^22^. In the previous studies, it was not determined whether the sustained efficacy of FK228 resulted from long residence time at HDACs versus alternate mechanisms (potentially involving HDAC expression levels, cellular accumulation of the reduced drug, inhibition of HDAC complexes and so forth). For prodrug molecules such as FK228, acellular assay formats are unsuitable to explore these mechanisms. The intracellular BRET technique was able to interrogate the dynamics of FK228 target engagement because of its compatibility with equilibrium and non-equilibrium binding analysis. Results presented here enabled a mechanistic interrogation into the enhanced phenotypic effect of FK228 under such pulse-treatment conditions, and indicate that HDAC occupancy is enhanced for this class of prodrug molecules under open system conditions. In a broader context, optimizing a lead compound for increased intracellular residence time at primary targets, while decreasing residence time at collateral targets, could mitigate potential drug side effects and increase safety profiles. The ability to configure the BRET assay to interrogate binding kinetics could be used to guide medicinal chemistry efforts during the workflow of lead optimization.

In summary, we have found that BRET can provide quantitative analysis of intracellular target engagement over a diverse set of target classes. This approach can be used to assess target engagement and binding kinetics in a simplified, homogeneous assay format. We have demonstrated that intracellular binding can be correlated with phenotypic potency, potentially allowing for primary drug targets to be differentiated from targets engaged collaterally. The use of BRET for interrogating compound binding to intracellular targets should therefore advance capabilities in drug design and mechanistic analysis.

## Methods

### Expression plasmid construction

To produce Nluc fusions with the targets interrogated in this study, pF31K Nluc [CMV/Neo] and pF32K [CMV/Neo] were used to place Nluc at the N-terminus or the C-terminus of the target protein (respectively) using the manufacturer's protocol (Promega). For N-terminal tagging of Nluc to the target protein in pF31K, the resulting fusion encoded a flexible Gly-Ser-Ser-Gly-Ala-Ile-Ala linker connecting Nluc with the target. For C-terminal tagging of Nluc to the target protein in pF32K, the resulting fusion encoded a flexible Val-Ser-Leu-Gly-Ser-Ser-Gly linker connecting the target protein with Nluc. Complementary DNAs encoded the following target proteins, and were a 100% match to their respective NCBI reference sequence identifiers; BRD2 (NP005095), BRD3 (AB383723.1), BRD4 (NP490597), BRDT (NP001229739), Kat2b (NP003875), HDAC1 (NP004955), HDAC2 (NP001518), HDAC3 (NP003874), HDAC4 (NP006028), HDAC5 (NP005465), HDAC6 (NP006035), HDAC7 (NP056216), HDAC8 (NP060956), HDAC9 (NP848510), HDAC10 (NP114408), HDAC11 (NP079103) and LCK (NP005347). To generate Nluc fusions with segregated domains of HDAC6, ORFs encoding amino acid residues 74-455 and residues 479-845 were used for HDAC6/CD1 and HDAC6/CD2, respectively. Nluc-HDAC6 CD2 was mutated at H610A/H611A (residues represented from full-lenth HDAC6) to generate a binding-deficient mutant protein. To generate Nluc fusions with segregated domains of BRD4, ORFs encoding amino acids 44-168 and residues 333-460 were used for BRD4/BD1 and BRD4/BD2, respectively.

### Cell transfection and BRET measurements under equilibrium conditions

To lower intracellular expression levels of the reporter fusion, Nluc/target fusion constructs into carrier DNA (pGEM3ZF-, Promega) at a mass ratio of 1:10 (mass/mass), before forming FuGENE HD complexes according to the manufacturer's protocol (Promega). DNA:FuGENE complexes were formed at a ratio of 1:3 (μg DNA per μl FuGENE). One part of the transfection complexes was then mixed with 20 parts (v/v) of HeLa cells (ATCC) suspended at a density of 2 × 10^5^ in DMEM (Gibco)+10% fetal bovine serum (FBS) (GE Healthcare), followed by incubation in a humidified, 37 °C/5% CO_2_ incubator for 20 h. Cells were trypsinized and resuspended in Opti-MEM without phenol red (Life Technologies). Cells were then seeded into white, nonbinding surface plates (Corning) at a density of 2 × 10^4^ cells/well. All chemical inhibitors were prepared as concentrated stock solutions in dimethylsulphoxide (DMSO) (Sigma-Aldrich). TDP-A was prepared by bacterial fermentation from the Cheng group, as described^19^,^41^. Remaining chemical inhibitors were purchased from Selleck Chemicals, with the exception of iBET, iBET-151, PFI-1 and CPI-203 (Xcessbio) and JQ-1 (EMD Biosciences). For generating tracer isotherms against targets expressed in cells, serially diluted tracer was added to cells in the presence or absence of 20 μM competing unlabelled compound (with the exception of BIBF-1120, for which Dasatinib was used). Cells were then equilibrated for 2 h before BRET measurements. For determining unlabelled compound isotherms, BRET tracers were added to the cells at fixed concentrations before test compound addition. A final concentration of 1 μM was used for SAHA-NCT and iBET-NCT. Experiments with BIBF1120-NCT utilized 3 μM. Serially diluted test compounds were then added to the cells and allowed to equilibrate for 2 h before BRET measurements. To measure BRET, NanoBRET NanoGlo Substrate-(Promega) was added, and filtered luminescence was measure on a BMG LABTECH Clariostar luminometer equipped with 450 nm BP filter (donor) and 610 nm LP filter (acceptor), using 0.5 s integration time with gain settings of 2,800 and 3,500, respectively. Background-corrected BRET ratios were determined by subtracting the BRET ratios of samples with excess competing ligand (20 μM) from the BRET ratios in the absence of competing ligand. Milli-BRET units (mBU) are the BRET values × 1,000. Apparent tracer affinity values were determined as described previously^13^ using curve fits in GraphPad Prism with the equation (equation (1)):


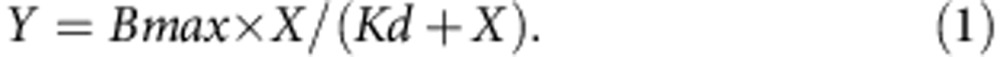


Competitive displacement data were then graphed with GraphPad Prism software using a three-parameter curve fit with the following equation (equation (2));


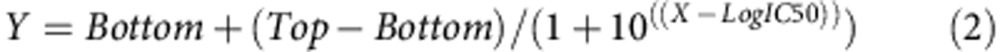


Normalized data were generated by assigning 100% to the theoretical maximum of the three-parameter curve fit and 0% for the theoretical minimum value of the three-parameter curve fit. Apparent affinity values for each compound were calculated from observed IC_50_ values according to the Cheng-Prusoff equationref. 39 (equation (3)):


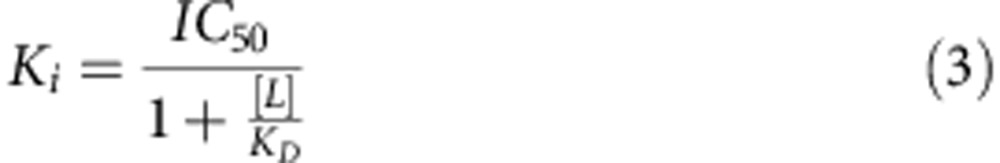


where [*L*] is the concentration of fluorescent ligand in μM and *K*_*D*_ is the apparent intracellular *K*_*D*_ of fluorescent ligand in μM. The apparent intracellular *K*_*D*_ values were calculated from the tracer saturation binding experiments described above.

### Kinetic analysis of target engagement via BRET

For kinetic analysis of the equilibration rates of SAHA, FK228 and TDP-A, cells transfected with Nluc-HDAC fusions as described above were pre-equilibrated for 2 h with 1 μM SAHA-NCT tracer prior addition of test compound. NanoBRET NanoGlo Substrate was added to the samples and equilibrated at room temperature for 30 min before BRET measurements. Immediately after addition of the unlabelled test compound, kinetic BRET analysis was performed at room temperature over the time indicated on a Thermo Varioskan Luminometer equipped with 450 nm BP filter (donor) and 610 nm LP filter (acceptor), using 0.5 s integration times.

### Kinetic analysis of ligand dissociation via BRET

For direct analysis of relative compound dissociation rates via BRET, 2 × 10^6^ cells transfected with HDAC1-Nluc (in 10 ml of Opti-MEM) were aliquoted into 15 ml conical tubes and first pre-equilibrated with DMSO (vehicle), SAHA (10 μM), mocetinostat (10 μM), FK228 (100 nM) or TDP-A (10 nM) for 3 h at 37 °C/5% CO_2_. Final concentration of DMSO was 0.1%. Cells treated with SAHA or mocetinostat were pelleted and resuspended in 10 mL of Opti-MEM in the presence of NanoBRET NanoGlo Substrate and 1 μM SAHA-NCT tracer immediately before BRET measurements. FK228 and TDP-A were subjected to an additional centrifugation and 10 ml wash step (with 5 min incubation) before BRET measurements, to ensure adequate removal of inhibitor from the cell medium. In addition to a zero-occupancy (DMSO/vehicle-treated) sample for determination of BRET_100_, a full-occupancy control sample (BRET_0_) was included, wherein 10 μM unmodified SAHA was left incubating on the cells (no washout) during the kinetic BRET analysis. To ensure that FK228 and TDP-A were not trapped within intact cells during the residence time analysis, a separate experiment was performed with digitonin added as lytic reagent (50 μg ml^−1^) added at the time of 1 μM SAHA-NCT tracer addition. BRET data were normalized to 100% signal from the positive control (defined as the maximum BRET value observed from cells treated with 1uM SAHA-NCT in the absence of unlabelled competing compound) and 0% signal (defined as the BRET value observed from cells treated with 1 uM SAHA-NCT+10 μM unmodified SAHA).

### BRET imaging

HeLa cells were transfected with Nluc/target fusion constructs as described previously (see above). Cells suspended at a density of 2 × 10^5^ cells per ml in DMEM (Gibco)+10% FBS (Hyclone) were plated into 35 mm tissue culture treated imaging dishes (ibidi) at 2 ml per dish and incubated 18–24 h at 37 °C/5% CO_2_. Media was removed from imaging dishes via gentle aspiration and replaced with 2 ml warm Opti-MEM without phenol red (Gibco) in the presence or absence of tracer +/− competing cold compound. Tracers and cold compound pairs used include 1 μM SAHA-NCT +/− 10 μM SAHA, 3 μM IBET-NCT +/− 10 μM IBET and 3 μM BIBF-1120-NCT +/− 10 μM Nilotinib. Cells were equilibrated for 2 h at 37 °C+5% CO_2_ before addition of NanoBRET NanoGlo Substrate. Images were captured on an Olympus LV200 microscope equipped with an environmental stage and a Hamamatsu ImagEM EMCCD camera. All images were acquired using a × 100/1.4 UPLanSApo objective and Olympus cellSens software. To image BRET events, images of donor and acceptor emission were acquired sequentially using a 460/80 bandpass filter and a 590 nm long-pass filter, respectively.

### Apoptosis and cell proliferation assays

To measure induction of apoptosis, HeLa cells were seeded into 96-well plates at a density of 10^4^ cells per well in DMEM+10% FBS. Serially diluted test compounds were added to the cells and incubated for 20 h. After incubation, an equal volume of Caspase-Glo (3/7) reagent (Promega) was added to the cells, and quantified on a GloMax-Multi Microplate Luminometer (Promega) according to the manufacturer's protocol. To measure inhibition of proliferation, HeLa cells were seeded into 96-well plates at a density of 2.5 × 10^3^ cells per well in DMEM+10% FBS. Serially diluted test compounds were added as described above and incubated 48 h. After incubation, an equal volume of CellTiter-Glo Luminescent Cell Viability Assay (Promega) was added to the cells, and luminescence was quantified according to the manufacturer's protocol. Data were then graphed with GraphPad Prism software as described above for the BRET target engagement experiments.

### Kinetic analysis of compound inhibition on purified HDAC1

Full-length human recombinant HDAC 1 was purchased from SignalChem. HDAC 1 enzymatic activity was measured using HDAC-Glo I/II Assay (Promega)^55^. To determine real-time IC_50_ values, 90 μl of 1/2,400 diluted HDAC 1 in HDAC-Glo I/II assay buffer was added to a opaque white, sterile, TC-treated 96-well assay plate (Corning). To enzyme, 100 μl of HDAC-Glo I/II detection reagent was added (190 μl total volume per well). The assay plate was mixed by a brief plate shake and the HDAC reaction was allowed to initiate and come to a steady-state luminescent signal by incubating for 20 min at room temperature. During this incubation, a serial dilution of SAHA, FK228, TDP-A was performed at a 200 × concentration in 100% DMSO in a separate master 96-well plate. A 10 μl aliquot of this master titration series was added to HDAC-Glo I/II assay buffer to generate a 20 × concentrated intermediate dilution containing 2.5 mM DTT for the prodrug inhibitors. Once the previous 20 min incubation was complete, 10 μl replicates (*n*=2 for SAHA and *n*=3 for FK228 and TDP-A) from this master intermediate titration series of compounds were transferred to the opaque white assay plate for a final total volume of 200 μl. The plate was then placed in a POLARstar OPTIMA (BMG LABTECH, Ortenberg, Germany) and luminescence was measured every 5 min for 120 min. For each time point, the raw luminescent signal for each test well of the appropriate compound was normalized to the no inhibition control well average (to calculate % inhibition). For each compound, the % inhibition was graphed versus inhibitor concentration for each time point to determine IC_50_. Data were then graphed with GraphPad Prism software as described above for the BRET target engagement experiments.

### Analysis of expression level of HDAC-NLuc fusions

HEK293 or HeLa cells were transfected with DNA constructs encoding HDAC1-NLuc at 0.8 μg ml^−1^ using PEI as transfection reagent as described previously^56^. To reduce expression levels the DNA was undiluted, diluted 1:10 and diluted 1:100 into a promoterless carrier DNA plasmid to generate a final total DNA concentration of 0.8 μg ml^−1^. Control cells were transfected with the promoterless carrier DNA plasmid only. Twenty-four hours after transfection the cells were collected using Cellstriper (Corning), washed with PBS and then lysed using detergent lysis buffer (mammalian lysis buffer from Promega) supplemented with 1:50 dilution of RQ1 DNase (Promega) and 1 × RQ1 DNAase buffer and 1 × protease inhibitor cocktail (Promega). 5% of each cell lysate was analysed by SDS-PAGE and electro-transferred onto a PVDF membrane (Life Technologies). The membrane was blocked for 1 h with 5% BSA (Promega) in TBS buffer and probed overnight at 4 °C with the primary antibody in TBS supplemented with 0.1% Tween-20 (TBST). After three washes in TBST, the membrane was incubated with a secondary HRP -conjugated antibody (Jackson laboratories) in TBST for 1 h, washed five times with TBST and one time with TBS. The immune-stained proteins were detected using enhanced chemiluminescent (ECL) reagent (Promega) and detected on the LAS400 imager (GE Healthcare). Antibody source: anti-HDAC1 (Abcam; ab46985), anti-HDAC2 (Abcam; ab51832).

### Activity of HDAC2 expressed in cell free expression system

HDAC2-NLuc, HDAC2 and NLuc were expressed in the S30 T7 High-Yield Protein expression system (Promega) as recommended by the manufacturer. A total of 40 μl of each expression reaction (in triplicates) were tested for HDAC2 activity using HDAC-Glo 2 assay (Promega). Luminescence was measured on a GloMAX luminometer (Promega). 

## Additional information

**How to cite this article:** Robers, M. B. *et al.* Target engagement and drug residence time can be observed in living cells with BRET. *Nat. Commun.* 6:10091 doi: 10.1038/ncomms10091 (2015).

## Supplementary Material

Supplementary Supplementary Figures 1-4, Supplementary Table 1-2, Supplementary Methods and Supplementary References

## References

[b1] Copeland R. A., Pompliano D. L. & Meek T. D. Drug-target residence time and its implications for lead optimization. Nat. Rev. Drug. Discov. 5, 730–739 (2006). 16888652 10.1038/nrd2082

[b2] Lu H. & Tonge P. J. Drug-target residence time: critical information for lead optimization. Curr. Opin. Chem. Biol. 14, 467–474 (2010). 20663707 10.1016/j.cbpa.2010.06.176 PMC2918722

[b3] Tummino P. J. & Copeland R. A. Residence time of receptor-ligand complexes and its effect on biological function. Biochemistry 47, 5481–5492 (2008). 18412369 10.1021/bi8002023

[b4] Bantscheff M. *et al.* Chemoproteomics profiling of HDAC inhibitors reveals selective targeting of HDAC complexes. Nat. Biotechnol. 29, 255–265 (2011). 21258344 10.1038/nbt.1759

[b5] Becher I. *et al.* Chemoproteomics reveals time-dependent binding of histone deacetylase inhibitors to endogenous repressor complexes. ACS Chem. Biol. 9, 1736–1746 (2014). 24877719 10.1021/cb500235n

[b6] Vauquelin G. & Charlton S. J. Exploring avidity: understanding the potential gains in functional affinity and target residence time of bivalent and heterobivalent ligands. Br. J. Pharmacol. 168, 1771–1785 (2013). 23330947 10.1111/bph.12106 PMC3623049

[b7] Vauquelin G. Rebinding: or why drugs may act longer *in vivo* than expected from their *in vitro* target residence time. Expert Opin. Drug Discov. 5, 927–941 (2010). 22823988 10.1517/17460441.2010.512037

[b8] Savitski M. M. *et al.* Proteomics. Tracking cancer drugs in living cells by thermal profiling of the proteome. Science 346, 1255784 (2014). 25278616 10.1126/science.1255784

[b9] Jafari R. *et al.* The cellular thermal shift assay for evaluating drug target interactions in cells. Nat. Protoc. 9, 2100–2122 (2014). 25101824 10.1038/nprot.2014.138

[b10] Martinez Molina D. *et al.* Monitoring drug target engagement in cells and tissues using the cellular thermal shift assay. Science 341, 84–87 (2013). 23828940 10.1126/science.1233606

[b11] Zwier J. M. *et al.* A fluorescent ligand-binding alternative using Tag-lite(R) technology. J. Biomol. Screen. 15, 1248–1259 (2010). 20974902 10.1177/1087057110384611

[b12] Lebakken C. S. *et al.* Development and applications of a broad-coverage, TR-FRET-based kinase binding assay platform. J. Biomol. Screen. 14, 924–935 (2009). 19564447 10.1177/1087057109339207

[b13] Stoddart L. A. *et al.* Application of BRET to monitor ligand binding to GPCRs. Nat. Methods. 12, 661–663 (2015). 26030448 10.1038/nmeth.3398 PMC4488387

[b14] Simon G. M., Niphakis M. J. & Cravatt B. F. Determining target engagement in living systems. Nat. Chem. Biol. 9, 200–205 (2013). 23508173 10.1038/nchembio.1211 PMC4004587

[b15] Moreau M. J., Morin I. & Schaeffer P. M. Quantitative determination of protein stability and ligand binding using a green fluorescent protein reporter system. Mol. Biosyst. 6, 1285–1292 (2010). 20454718 10.1039/c002001j

[b16] Taipale M. *et al.* Chaperones as thermodynamic sensors of drug-target interactions reveal kinase inhibitor specificities in living cells. Nat. Biotechnol. 31, 630–637 (2013). 23811600 10.1038/nbt.2620 PMC3774174

[b17] Savitski M. M. *et al.* Tracking cancer drugs in living cells by thermal profiling of the proteome. Science 346, 1255784 (2014). 25278616 10.1126/science.1255784

[b18] Furumai R. *et al.* FK228 (depsipeptide) as a natural prodrug that inhibits class I histone deacetylases. Cancer Res. 62, 4916–4921 (2002). 12208741

[b19] Wang C. *et al.* Thailandepsins: bacterial products with potent histone deacetylase inhibitory activities and broad-spectrum antiproliferative activities. J. Nat. Prod. 74, 2031–2038 (2011). 21793558 10.1021/np200324x PMC3204160

[b20] Giang I., Boland E. L. & Poon G. M. Prodrug applications for targeted cancer therapy. AAPS J. 16, 899–913 (2014). 25004822 10.1208/s12248-014-9638-z PMC4147050

[b21] Wilson A. J., Cheng Y. Q. & Khabele D. Thailandepsins are new small molecule class I HDAC inhibitors with potent cytotoxic activity in ovarian cancer cells: a preclinical study of epigenetic ovarian cancer therapy. J. Ovarian Res. 5, 12 (2012). 22531354 10.1186/1757-2215-5-12 PMC3394212

[b22] Wei D. G. *et al.* Histone deacetylase inhibitor romidepsin induces HIV expression in CD4 T cells from patients on suppressive antiretroviral therapy at concentrations achieved by clinical dosing. PLoS Pathog. 10, e1004071 (2014). 24722454 10.1371/journal.ppat.1004071 PMC3983056

[b23] Crabb S. J. *et al.* Characterisation of the *in vitro* activity of the depsipeptide histone deacetylase inhibitor spiruchostatin A. Biochem. Pharmacol. 76, 463–475 (2008). 18611394 10.1016/j.bcp.2008.06.004

[b24] Ito T. *et al.* Real-time imaging of histone H4K12-specific acetylation determines the modes of action of histone deacetylase and bromodomain inhibitors. Chem. Biol. 18, 495–507 (2011). 21513886 10.1016/j.chembiol.2011.02.009

[b25] Machleidt T. *et al.* NanoBRET-A novel BRET platform for the analysis of protein-protein interactions. ACS Chem. Biol. 10, 1797–1804 (2015). 26006698 10.1021/acschembio.5b00143

[b26] Pfleger K. D. & Eidne K. A. Illuminating insights into protein-protein interactions using bioluminescence resonance energy transfer (BRET). Nat. Methods 3, 165–174 (2006). 16489332 10.1038/nmeth841

[b27] Pfleger K. D., Seeber R. M. & Eidne K. A. Bioluminescence resonance energy transfer (BRET) for the real-time detection of protein-protein interactions. Nat. Protoc. 1, 337–345 (2006). 17406254 10.1038/nprot.2006.52

[b28] Dacres H., Dumancic M. M., Horne I. & Trowell S. C. Direct comparison of bioluminescence-based resonance energy transfer methods for monitoring of proteolytic cleavage. Anal. Biochem. 385, 194–202 (2009). 19026607 10.1016/j.ab.2008.10.040

[b29] Friedman Ohana R. *et al.* Deciphering the cellular targets of bioactive compounds using a chloroalkane capture tag. ACS Chem. Biol. 10, 2316–2324 (2015). 26162280 10.1021/acschembio.5b00351

[b30] Griss R. *et al.* Bioluminescent sensor proteins for point-of-care therapeutic drug monitoring. Nat. Chem. Biol. 10, 598–603 (2014). 24907901 10.1038/nchembio.1554

[b31] Hall M. P. *et al.* Engineered luciferase reporter from a deep sea shrimp utilizing a novel imidazopyrazinone substrate. ACS Chem. Biol. 7, 1848–1857 (2012). 22894855 10.1021/cb3002478 PMC3501149

[b32] Bradner J. E. *et al.* Chemical phylogenetics of histone deacetylases. Nat. Chem. Biol. 6, 238–243 (2010). 20139990 10.1038/nchembio.313 PMC2822059

[b33] Finnin M. S. *et al.* Structures of a histone deacetylase homologue bound to the TSA and SAHA inhibitors. Nature 401, 188–193 (1999). 10490031 10.1038/43710

[b34] Wang D. F., Helquist P., Wiech N. L. & Wiest O. Toward selective histone deacetylase inhibitor design: homology modeling, docking studies, and molecular dynamics simulations of human class I histone deacetylases. J. Med. Chem. 48, 6936–6947 (2005). 16250652 10.1021/jm0505011

[b35] Miller K. M. *et al.* Human HDAC1 and HDAC2 function in the DNA-damage response to promote DNA nonhomologous end-joining. Nat. Struct. Mol. Biol. 17, 1144–1151 (2010). 20802485 10.1038/nsmb.1899 PMC3018776

[b36] Yao Y. L. & Yang W. M. Beyond histone and deacetylase: an overview of cytoplasmic histone deacetylases and their nonhistone substrates. J. Biomed. Biotechnol. 2011, 146493 (2011). 21234400 10.1155/2011/146493 PMC3014693

[b37] Zhang Y., Gilquin B., Khochbin S. & Matthias P. Two catalytic domains are required for protein deacetylation. J. Biol. Chem. 281, 2401–2404 (2006). 16272578 10.1074/jbc.C500241200

[b38] Zou H., Wu Y., Navre M. & Sang B. C. Characterization of the two catalytic domains in histone deacetylase 6. Biochem. Biophys. Res. Commun. 341, 45–50 (2006). 16412385 10.1016/j.bbrc.2005.12.144

[b39] Cheng Y. & Prusoff W. H. Relationship between the inhibition constant (K1) and the concentration of inhibitor which causes 50 per cent inhibition (I50) of an enzymatic reaction. Biochem. Pharmacol. 22, 3099–3108 (1973). 4202581 10.1016/0006-2952(73)90196-2

[b40] Witt O., Deubzer H. E., Milde T. & Oehme I. HDAC family: what are the cancer relevant targets? Cancer. Lett. 277, 8–21 (2009). 18824292 10.1016/j.canlet.2008.08.016

[b41] Wang C., Flemming C. J. & Cheng Y. Q. Discovery and activity profiling of thailandepsins A through F, potent histone deacetylase inhibitors, from E264. MedChemComm 3, 976–981 (2012). 23997923 10.1039/C2MD20024D PMC3755959

[b42] Wilting R. H. *et al.* Overlapping functions of Hdac1 and Hdac2 in cell cycle regulation and haematopoiesis. EMBO J. 29, 2586–2597 (2010). 20571512 10.1038/emboj.2010.136 PMC2928690

[b43] Yamaguchi T. *et al.* Histone deacetylases 1 and 2 act in concert to promote the G1-to-S progression. Genes Dev. 24, 455–469 (2010). 20194438 10.1101/gad.552310 PMC2827841

[b44] Neumann L., von Konig K. & Ullmann D. HTS reporter displacement assay for fragment screening and fragment evolution toward leads with optimized binding kinetics, binding selectivity, and thermodynamic signature. Methods Enzymol. 493, 299–320 (2011). 21371596 10.1016/B978-0-12-381274-2.00012-1

[b45] Di Micco S. *et al.* Structural basis for the design and synthesis of selective HDAC inhibitors. Bioorg. Med. Chem. 21, 3795–3807 (2013). 23693069 10.1016/j.bmc.2013.04.036

[b46] Fournel M. *et al.* MGCD0103, a novel isotype-selective histone deacetylase inhibitor, has broad spectrum antitumor activity *in vitro* and *in vivo*. Mol. Cancer. Ther. 7, 759–768 (2008). 18413790 10.1158/1535-7163.MCT-07-2026

[b47] Bonfils C. *et al.* Evaluation of the pharmacodynamic effects of MGCD0103 from preclinical models to human using a novel HDAC enzyme assay. Clin. Cancer Res. 14, 3441–3449 (2008). 18519775 10.1158/1078-0432.CCR-07-4427 PMC3444140

[b48] Schulze J. *et al.* Cell-based protein stabilization assays for the detection of interactions between small-molecule inhibitors and BRD4. J Biomol. Screen. 20, 180–189 (2014). 25266565 10.1177/1087057114552398

[b49] Davis M. I. *et al.* Comprehensive analysis of kinase inhibitor selectivity. Nat. Biotechnol. 29, 1046–1051 (2011). 22037378 10.1038/nbt.1990

[b50] Karaman M. W. *et al.* A quantitative analysis of kinase inhibitor selectivity. Nat. Biotechnol. 26, 127–132 (2008). 18183025 10.1038/nbt1358

[b51] Guenther M. G., Barak O. & Lazar M. A. The SMRT and N-CoR corepressors are activating cofactors for histone deacetylase 3. Mol. Cell. Biol. 21, 6091–6101 (2001). 11509652 10.1128/MCB.21.18.6091-6101.2001 PMC87326

[b52] Couturier C. & Deprez B. Setting Up a bioluminescence resonance energy transfer high throughput screening assay to search for protein/protein interaction inhibitors in mammalian cells. Front. Endocrinol. 3, 100 (2012). 10.3389/fendo.2012.00100 PMC3438444 22973258

[b53] Dacres H., Dumancic M. M., Horne I. & Trowell S. C. Direct comparison of fluorescence- and bioluminescence-based resonance energy transfer methods for real-time monitoring of thrombin-catalysed proteolytic cleavage. Biosens. Bioelectron. 24, 1164–1170 (2009). 18723336 10.1016/j.bios.2008.07.021

[b54] Lebakken C. S., Reichling L. J., Ellefson J. M. & Riddle S. M. Detection of allosteric kinase inhibitors by displacement of active site probes. J Biomol. Screen. 17, 813–821 (2012). 22453235 10.1177/1087057112439889

[b55] Halley F. *et al.* A bioluminogenic HDAC activity assay: validation and screening. J Biomol. Screen. 16, 1227–1235 (2011). 21832257 10.1177/1087057111416004

[b56] Ohana R. F. *et al.* HaloTag-based purification of functional human kinases from mammalian cells. Protein Expr. Purif. 76, 154–164 (2011). 21129486 10.1016/j.pep.2010.11.014

