# Supplementary material for: Target engagement and drug residence time can be observed in living cells with BRET
Source: Nat Commun. 2015 Dec 3;6:10091. doi: 10.1038/ncomms10091 (PMC4686764; doi:10.1038/ncomms10091)
Supplement: Supplementary — Figures 1-4, Supplementary Table 1-2, Supplementary Methods and Supplementary References [file ncomms10091-s1.pdf]

Supplementary Figures

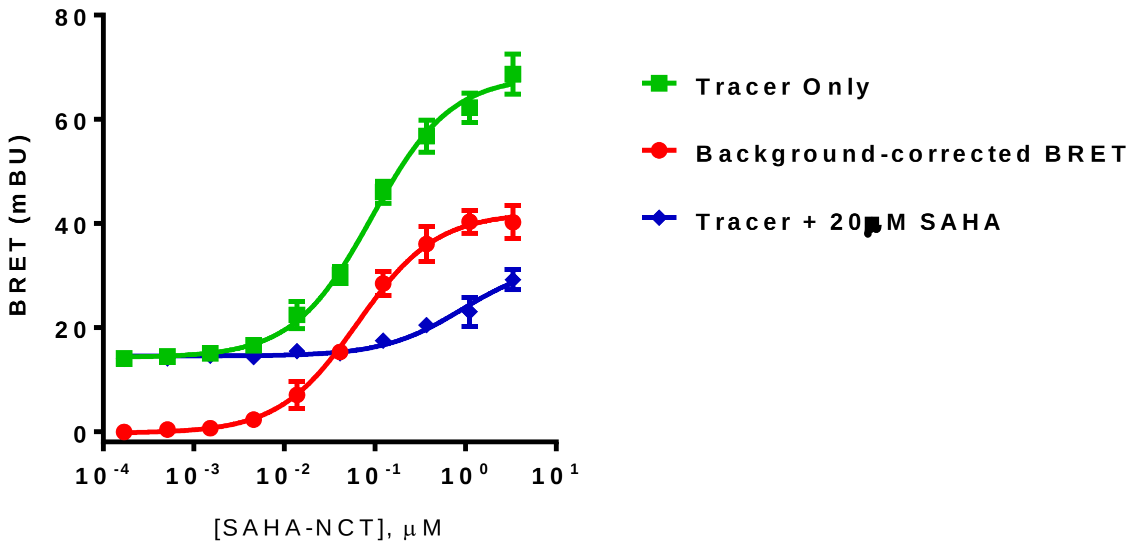

Supplementary Fig. 1a

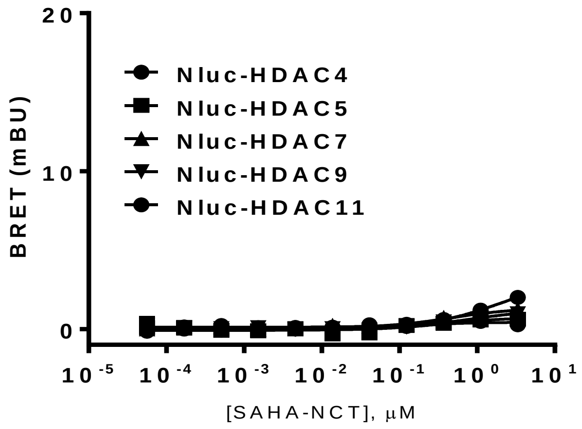

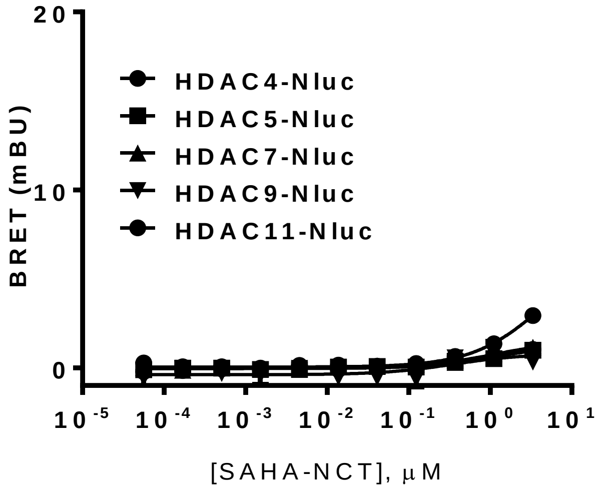

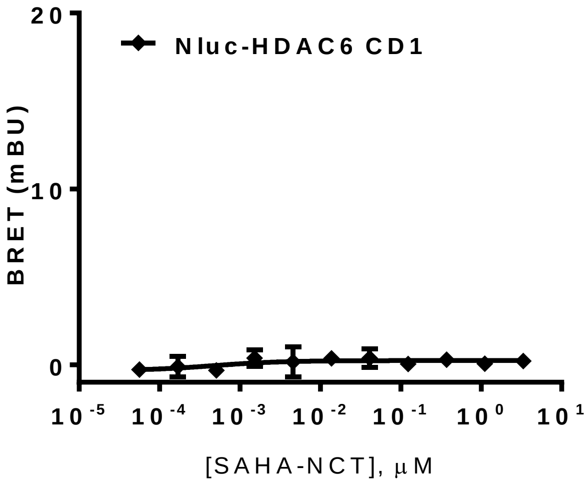

Supplementary Fig. 1b

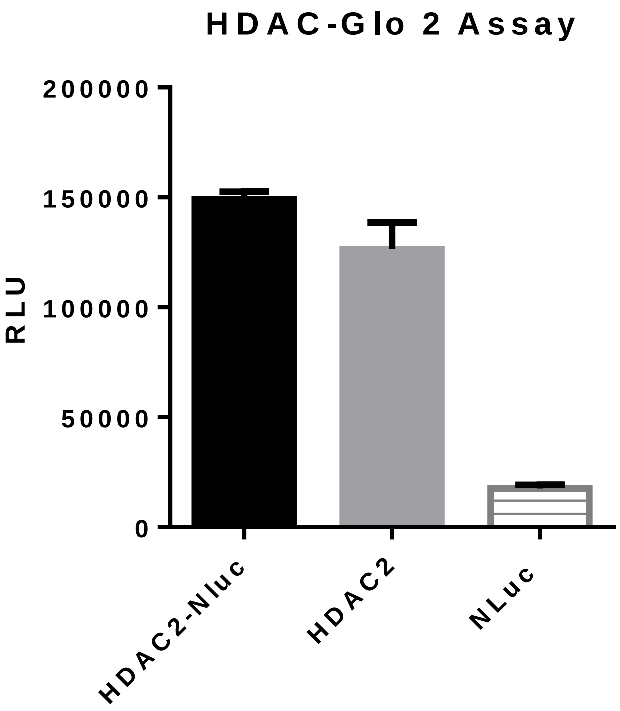

Supplementary Fig. 1c

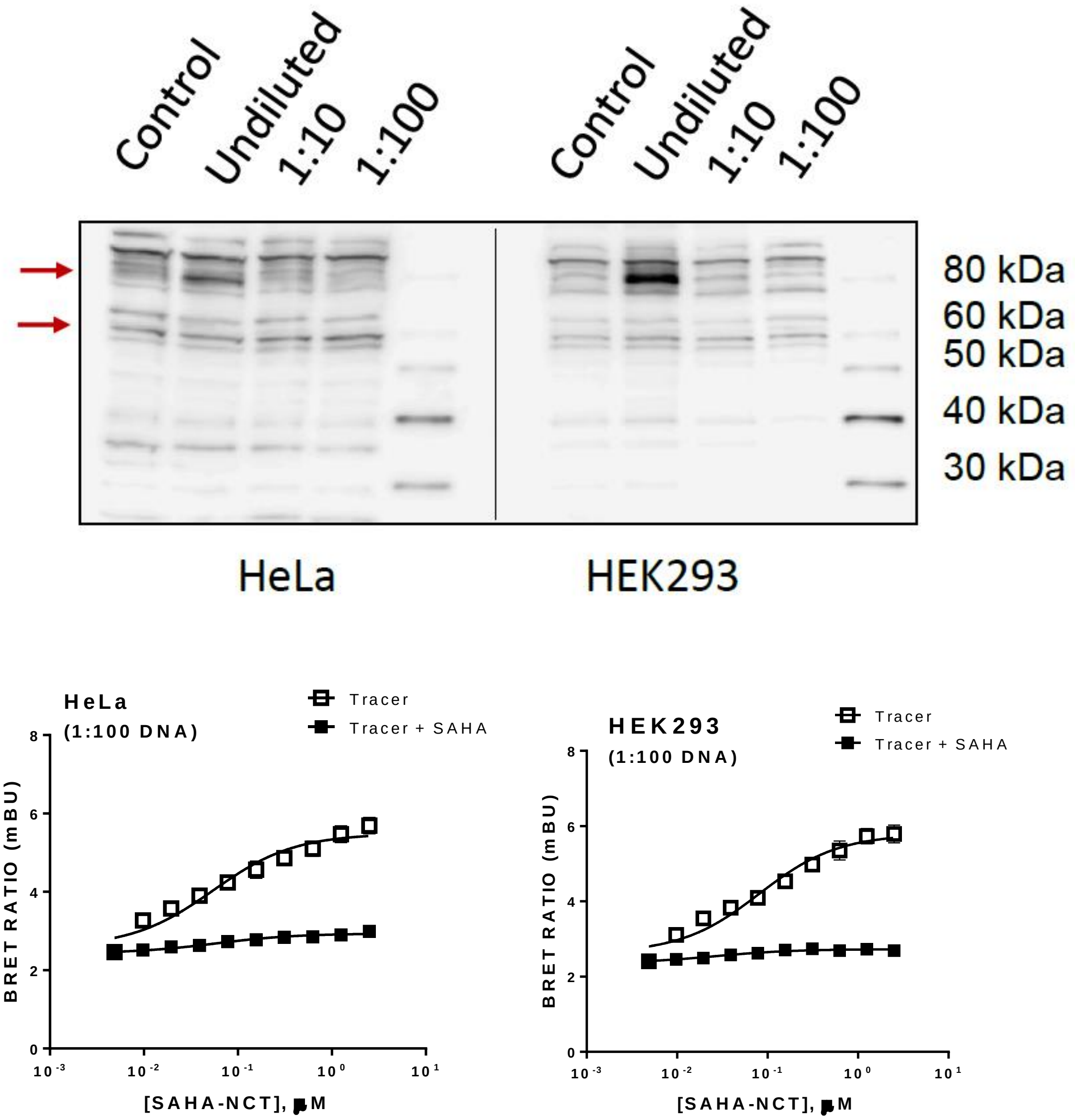

Supplementary Fig. 1d

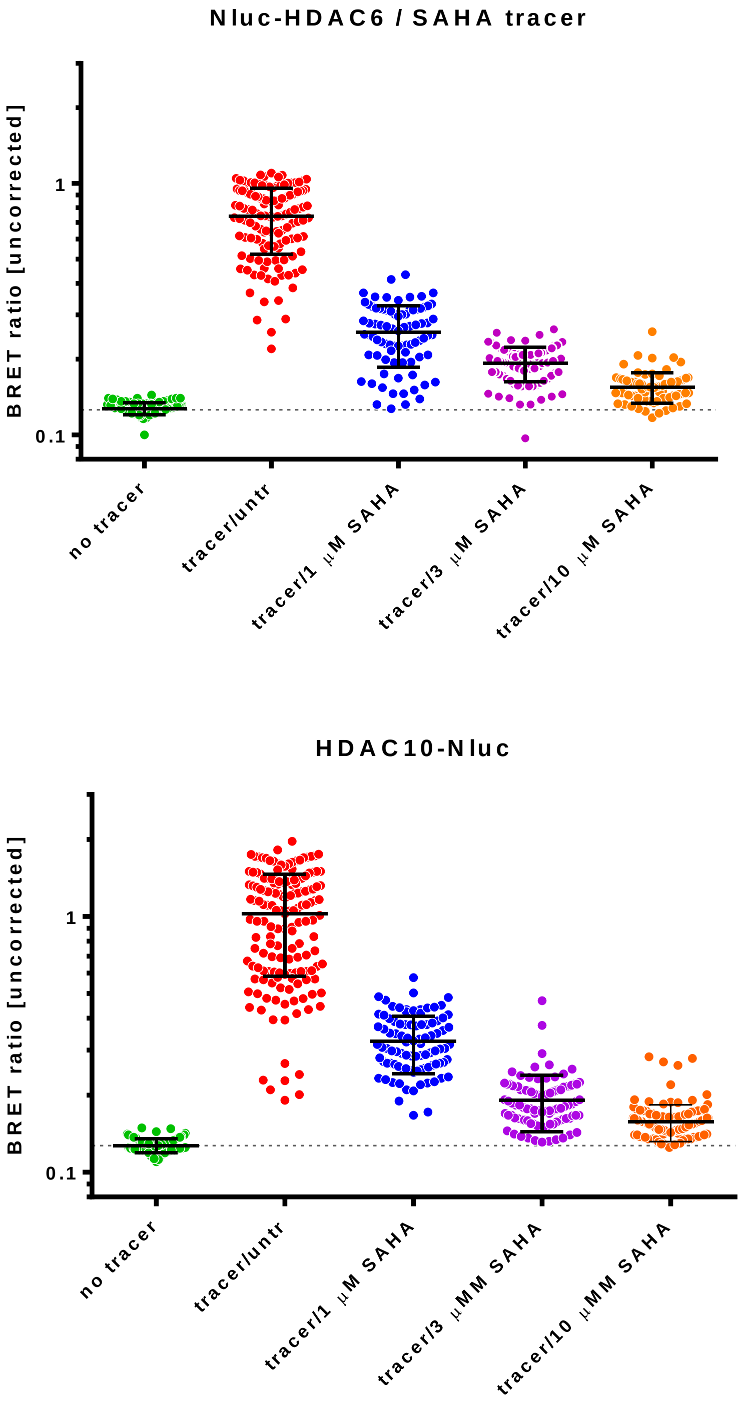

Supplementary Fig. 1e

Supplementary Figure 1. Measuring target engagement at HDACs using intracellular BRET complexes. (a) Representative intracellular target engagement data at Nluc-HDAC6, demonstrating the concentration-dependent BRET signal generated by the SAHA-NCT tracer in the presence or absence of 20 μM of competing unlabeled SAHA. Resulting background-corrected BRET signal is also shown in the graph. To separate the specific BRET signal from spurious signal which arises at higher tracer concentrations, the BRET values at each concentration were corrected by subtracting values from parallel samples that further contained an excess of unmodified compound (i.e., 20 μM SAHA). The presence of excess compound competitively displaces the tracer from the target, so that the corrected BRET values represent energy transfer produced specifically from the bound tracer. (b) Compared to Class I and Class IIb HDACs, small BRET signal is observed between the SAHA-NCT tracer and Class IIa HDACs (HDACs 4, 5, 7, and 9), catalytic domain 1 of HDAC6, and the Class IV HDAC11. Data are mean ± SD. of four data points. (c) Confirmation of HDAC activity of HDAC2-Nluc fusions. Both HDAC2 (untagged) and HDAC2-Nluc were expressed in a cell-free expression system (S30) and subjected to HDAC-Glo-2 activity assays. Both untagged HDAC2 and HDAC2-Nluc showed activity compared to control samples without HDAC2-encoded DNA. (d) Top: Western analysis, using anti-HDAC1 antibody, indicates that transient transfection using HDAC1-Nluc DNA diluted either 1:10 or 1:100 into promoterless carrier DNA results in HDAC1 levels below endogenous levels of HDAC1 in both HeLa (left) and HEK293 (right). Bottom: BRET is able to measure target engagement at HDAC1, in samples transfected with 1:100 dilutions of HDAC1-Nluc DNA, corresponding to protein levels below that of endogenously expressed HDAC1 in both HeLa (left) and HEK293 cells (right). (e) Single cell analysis of BRET between the SAHA-NCT tracer and Nluc-HDAC6 or HDAC10-Nluc within HeLa. BRET was measured on an LV200 bioluminescence microscope, and each data point on the histogram represents the BRET signal from an individual cell. The mean BRET signal is indicated, ± S.D.

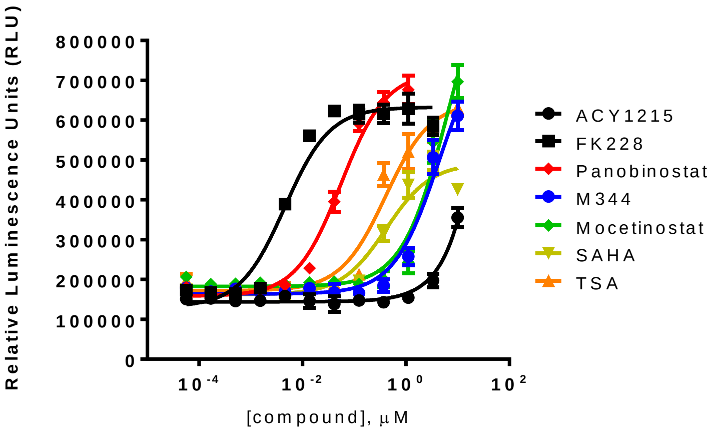

Supplementary Fig. 2a

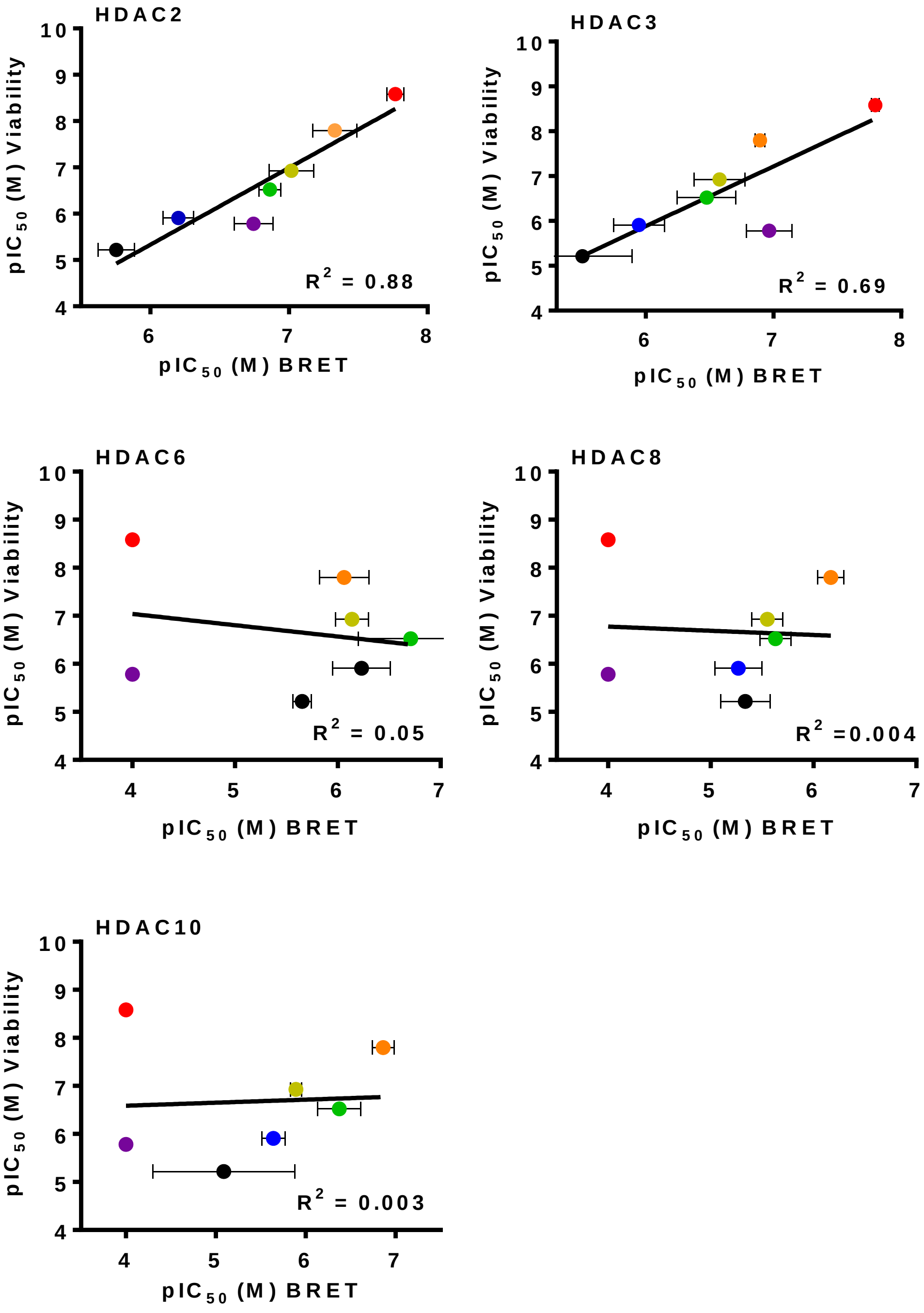

Supplementary Fig. 2b

Supplementary Figure 2. (a) Rank-order potency of an HDAC inhibitor panel for induction of apoptosis in HeLa cells. 24 hour treatment of HeLa cells with the HDAC inhibitor panel results in a range of potencies of apoptosis induction, as measured by activation of caspase 3/7 activity. Data are the mean of 4 data points ± S.D. (b) Correlation of anti-proliferative potency of HDAC inhibitors, as determined by cellular ATP levels in HeLa cells, with HDAC isozyme affinity. Compounds such as mocetinostat or FK228 having an IC_{50} value of >10 uM by competitive displacement, were assigned a pIC_{50} of 4 in the analysis (Supplemental Table 2). Data are mean of 3 independent experiments ± S.D.

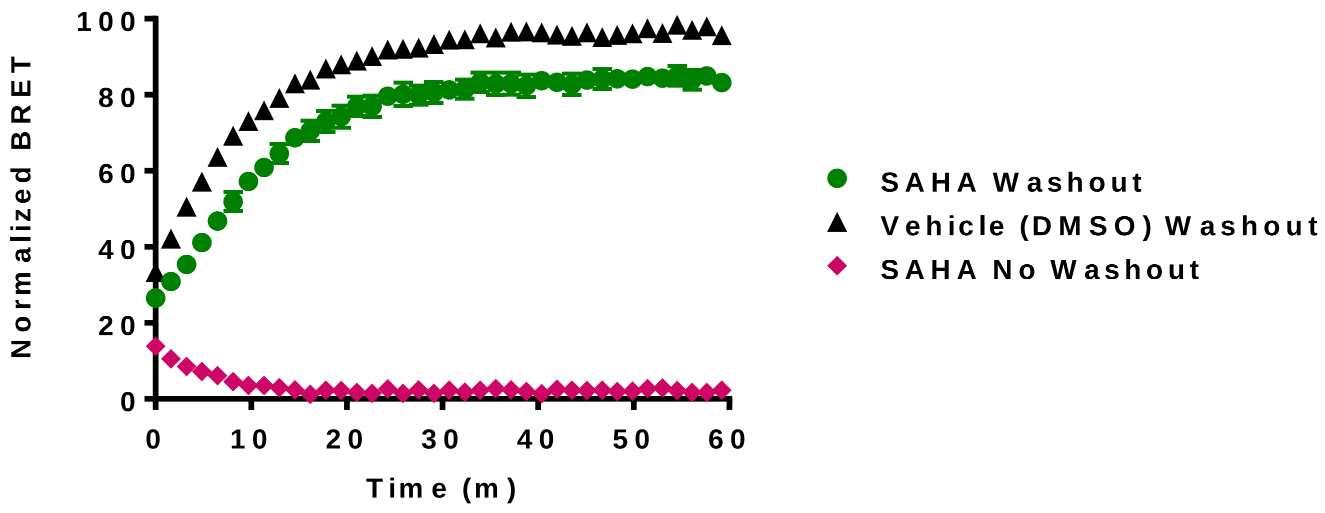

Supplementary Fig 3a

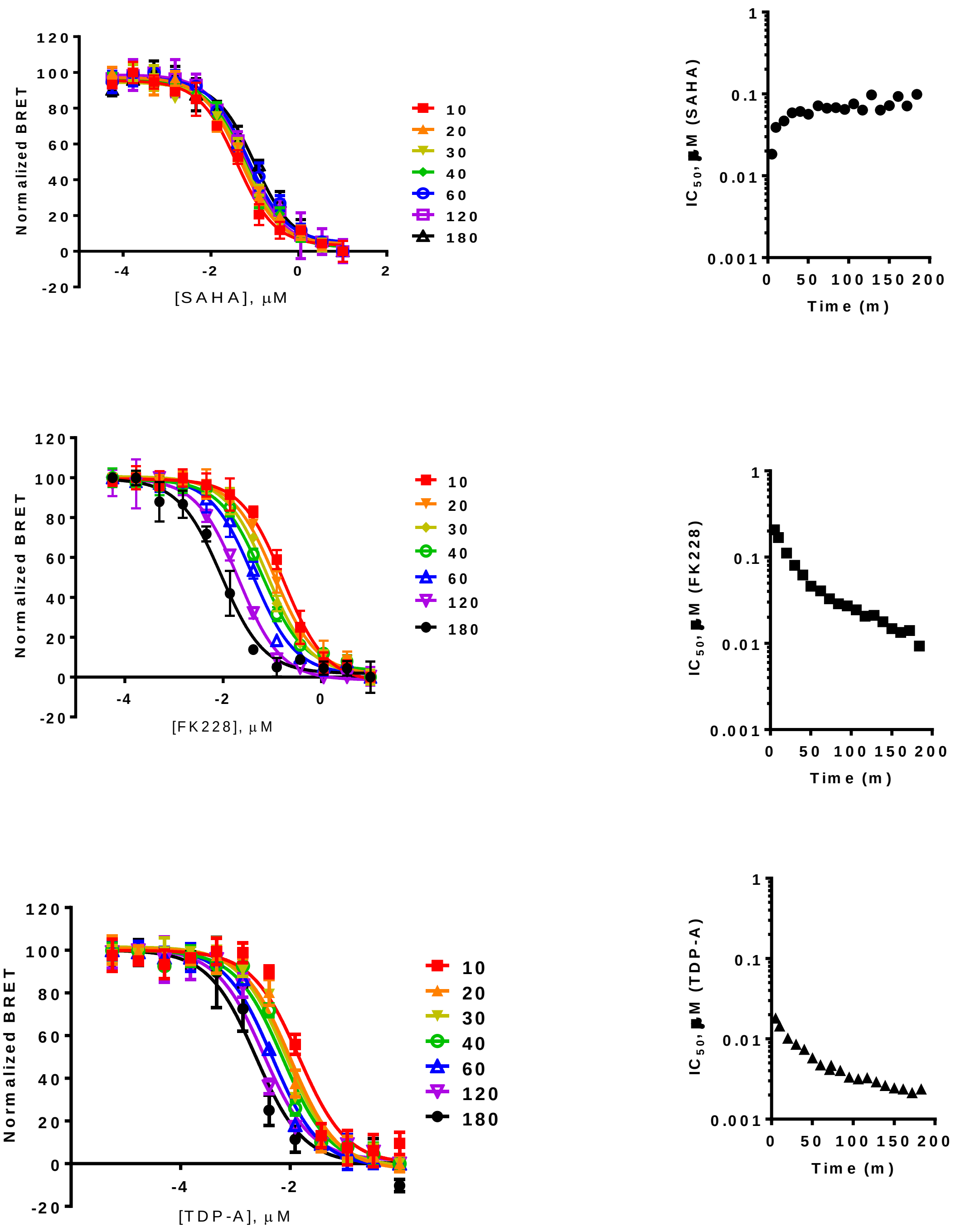

Supplementary Fig. 3b

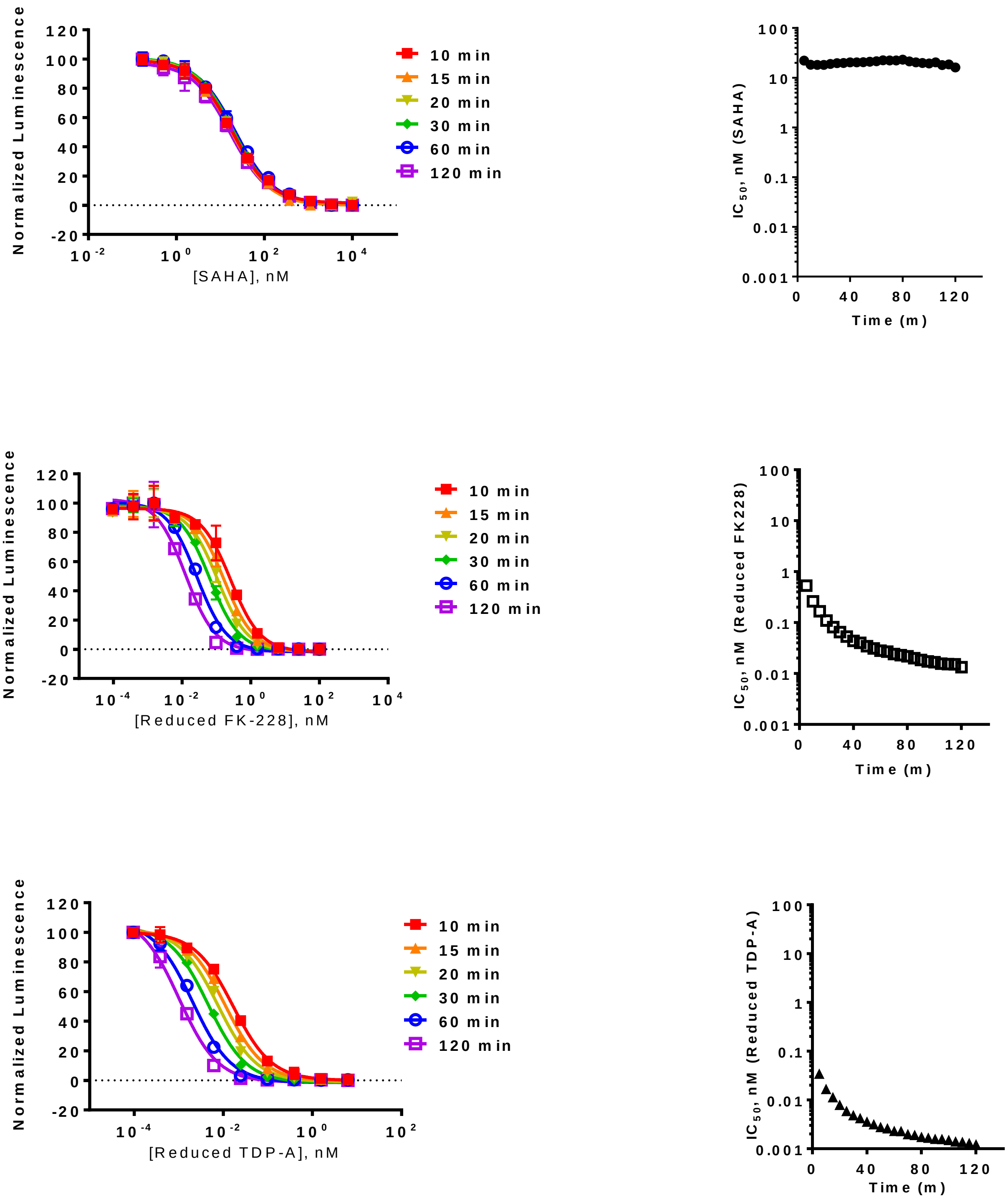

Supplementary Fig. 3c

1. Saturate w/ test ligand at approximate [EC_{90}], as determined from previous target engagement experiments under equilibrium conditions. This treatment should be performed for 2 hours to ensure target occupancy

2. Perform washes to ensure ligand removal

3. Add 1 μM tracer and NanoLuc Substrate

4. Immediately measure BRET (real time)

5. Optional: Include digitonin as a control for intracellular trapping of test ligand

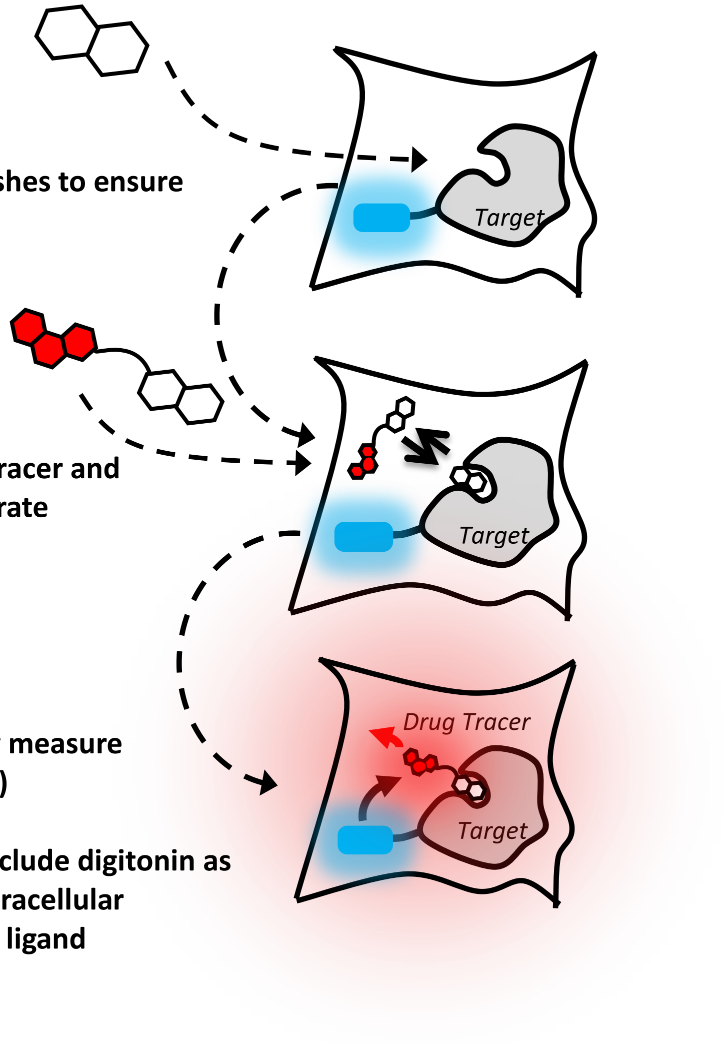

Supplementary Fig. 3d

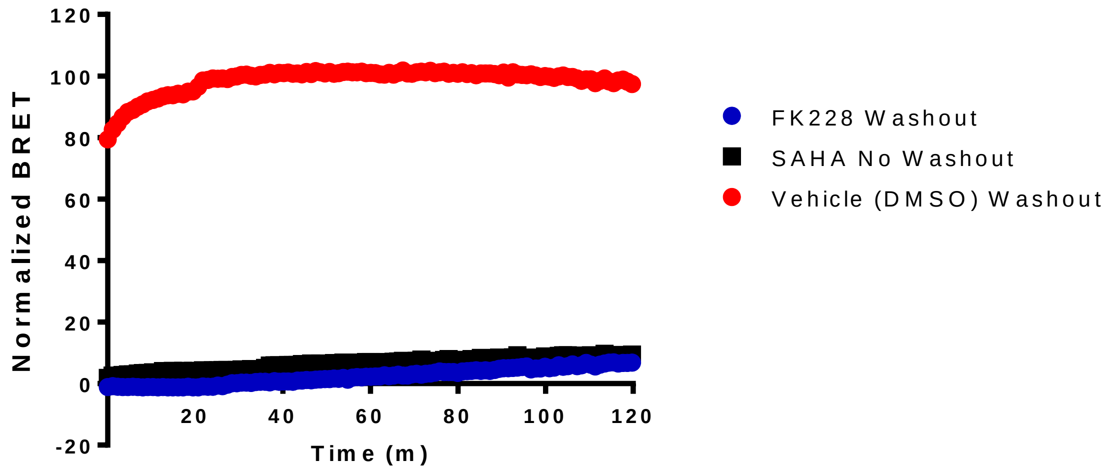

Supplementary Fig. 3e

Supplementary Figure 3. Characterization of HDAC1 tracer binding and enzymatic inhibition at HDAC1. (a) Kinetic BRET analysis revealed the rate of formation of intracellular BRET complexes between HDAC1-Nluc and the SAHA-NCT tracer in HeLa cells. Observed association kinetics of SAHA-NCT at HDAC1-Nluc after washout of vehicle (DMSO) were fast, reaching a plateau in approximately 20 min. BRET complexes were inhibited in the presence of 10 μM unlabeled SAHA, representing a full occupancy (zero dissociation) control. Washout of SAHA showed a kinetic trace similar to vehicle (DMSO) treated cells, indicating fast dissociation kinetics of SAHA as expected. Data are the mean ± S.E.M. of four independent experiments (b) Measuring the differential rates of binding of SAHA, FK228, and TDP-A to HDAC1 in live HeLa cells using BRET. SAHA binding kinetics were rapid, while a kinetic lag was observed for the prodrug inhibitors FK228 and TDP-A. Data represent the mean ± S.D. of three data points for the prodrug inhibitors and two data points for SAHA. (c) Enzymatic inhibition of purified HDAC1 by SAHA, FK228, and TDP-A. SAHA inhibition kinetics were rapid, while a kinetic lag is observed for the prodrug inhibitors FK228 and TDP-A. Data were graphed as activity versus compound dose (left panels) and IC_{50} versus time (right panels). Data are mean ± S.D. of three data points for the prodrug inhibitors and

two data points for SAHA. (d) Schematic of protocol for residence time analysis at HDAC1. (e) Measuring the intracellular residence time of HDAC inhibitors at HDAC1 following inhibitor washout and under lytic conditions. Residence time analysis by BRET (1 μM SAHA-NCT tracer + digitonin added at time = 0) reveals remarkably slow dissociation rates for FK228 (blue) in cell lysates. Occupancy of FK228 under non-equilibrium conditions was indistinguishable from the samples with a saturating dose (20μM) of SAHA under non-washout conditions (black). Data are mean ± S.D. of three data points.

Supplementary Fig. 4a

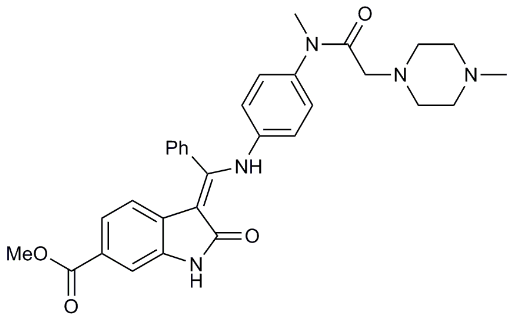

BIBF 1120

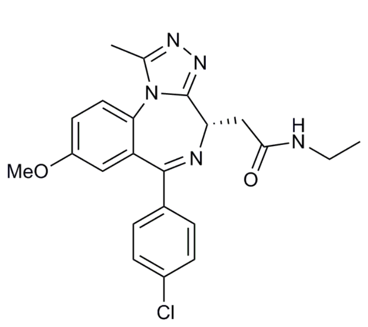

iBET 762

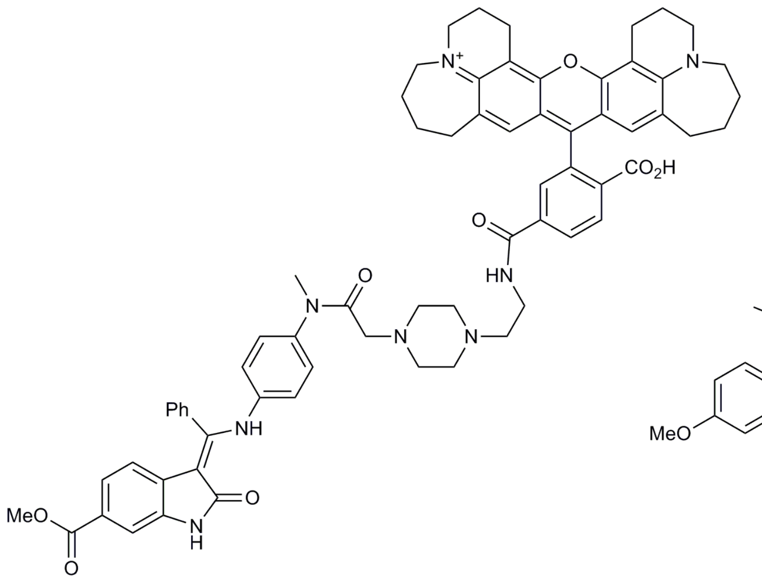

BIBF-NCT

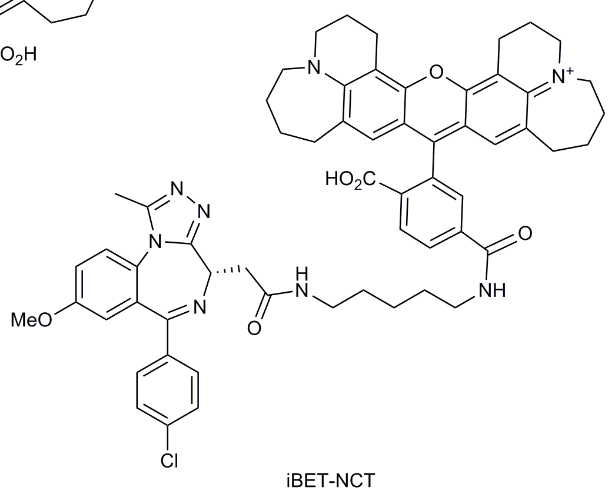

iBET-NCT

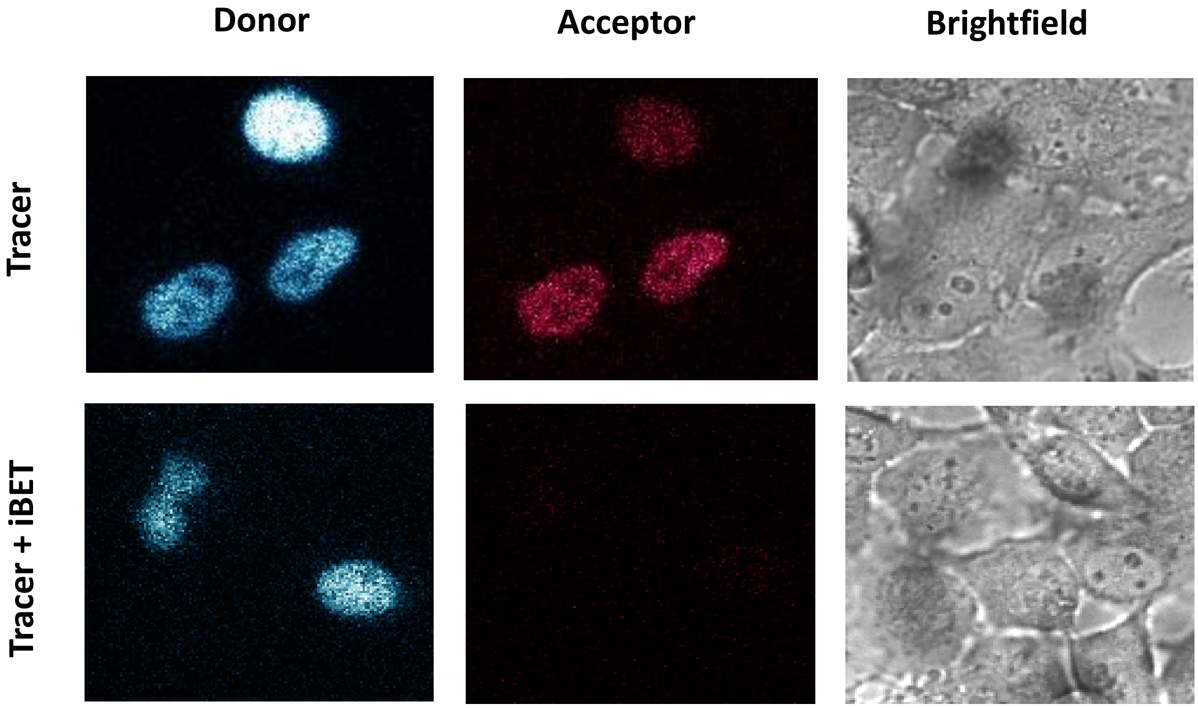

Supplementary Fig. 4b

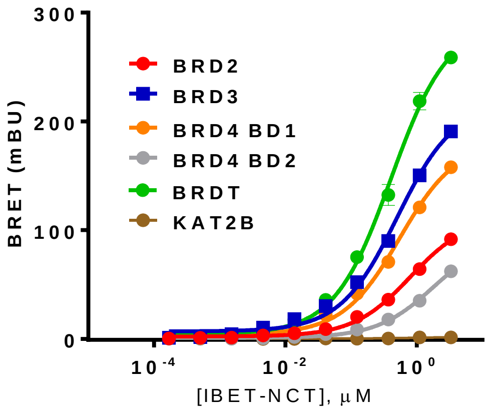

Supplementary Fig. 4c

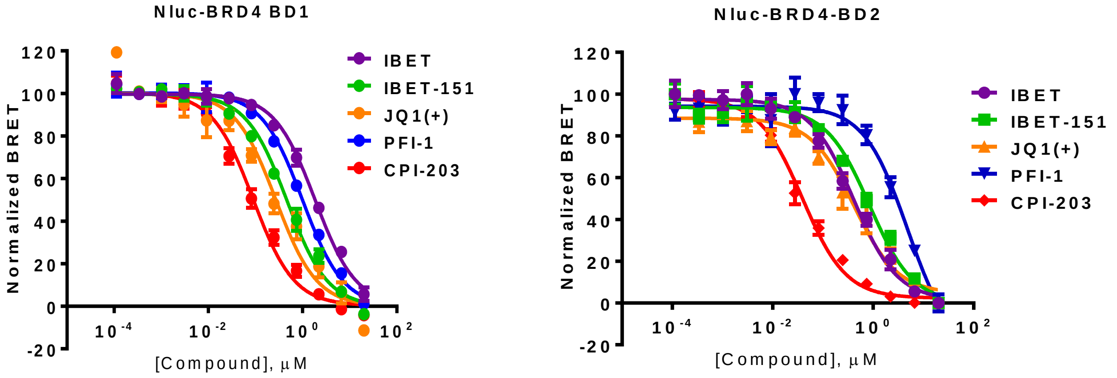

Supplementary Fig. 4d

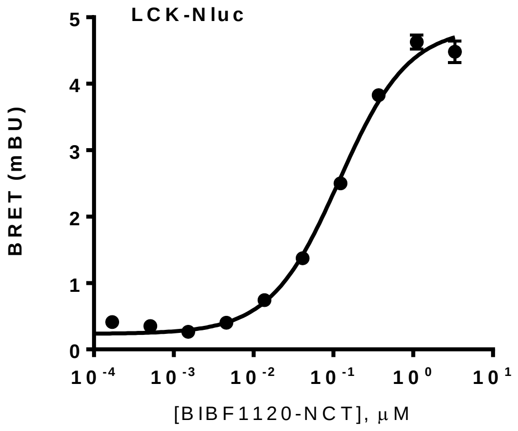

Supplementary Fig. 4e

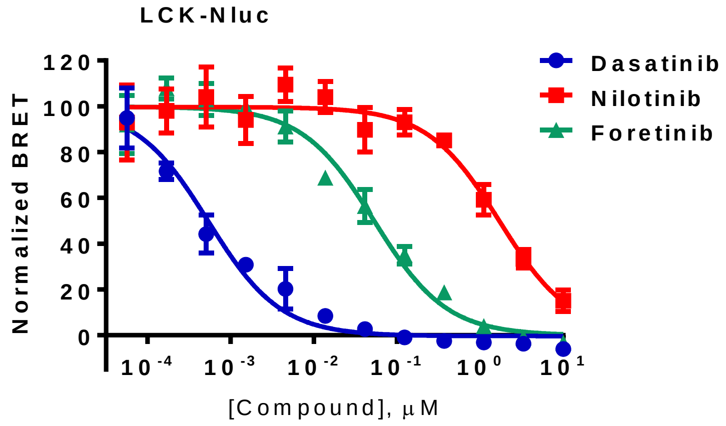

Supplementary Fig. 4f

Supplementary Figure 4. Measuring intracellular target engagement at bromodomains (BRDs) and the LCK kinase using BRET. (a) Chemical structures of the kinase tracer derived from BIBF1120 and NCT dye (left), as well as the structure of the bromodomain tracer derived from iBET762 and NCT dye (right). (b) BRET imaging demonstrates specific intracellular BRET signal between the iBET-NCT tracer (2 μM) and fusion of Nluc with full-length BRD4. Addition of a molar excess (10 μM) of unlabeled iBET762 results in attenuation of the BRET signal, indicating signal specificity. (c) Specific and dose-dependent BRET was

observed with Nluc-fusions to BET family members BRD2, BRD3, BRDT, and the segregated ligand binding domains of BRD4. Negligible BRET was observed for Kat2b serving as a negative control. BRET values at each tracer concentration were background-corrected by parallel measurements made in the presence of an excess of unmodified compound (20 μM) (described in Methods). (d) Use of BRET to measure rank-order affinity of BRD inhibitor panel against segregated domains of BRD4 within live HeLa cells (1 μM IBET-NCT held constant). (e) A kinase tracer derived from BIBF1120 and NCT dye engaged intracellular LCK fused to Nluc, resulting in specific, concentration-dependent BRET. BRET values at each tracer concentration were background-corrected by parallel measurements made in the presence of an excess of unmodified compound (20 μM) (described in Methods). (f) BRET measurements showing the relative affinity of LCK inhibitors in HeLa cells (1 μM BIBF1120-NCT). Data are mean ± S.D. of four data points.

Pharmacological Profile of SAHA-NCT and SAHA			
**Isotype**	**Tracer Apparent K_d, nM**	**IC_{50}, nM SAHA**	**K_i, nM SAHA**
**HDAC1**	**300**	**81**	**17**
**HDAC2**	**210**	**150**	**26**
**HDAC3**	**1500**	**380**	**230**
**HDAC6**	**79**	**170**	**12**
**HDAC8**	**810**	**1700**	**760**
**HDAC10**	**77**	**310**	**22**

Supplementary Table 1. Intracellular target engagement profile of SAHA-NCT and SAHA using BRET. Values are means of 3 independent experiments.

	Pharmacological Profile of Selected HDAC Inhibitors, pIC50 (M) ± S.D.						
**Compound**	**HDAC1**	**HDAC2**	**HDAC3**	**HDAC6**	**HDAC8**	**HDAC10**	**Viability**
**ACY1215**	6.1 ± 0.03	5.8 ± 0.13	5.5 ± 0.39	5.7 ± 0.09	5.3 ± 0.24	5.1 ± 0.79	5.2 ± 0.11
**FK228**	7.9 ± 0.15	7.8 ± 0.06	7.8 ± 0.03	<4	<4	<4	8.6 ± 0.10
**panobinostat**	7.6 ± 0.13	7.3 ± 0.16	6.9 ± 0.04	6.1 ± 0.24	6.2 ± 0.13	6.9 ± 0.12	7.8 ± 0.016
**M344**	6.6 ± 0.02	6.2 ± 0.11	5.9 ± 0.20	6.2 ± 0.28	5.3 ± 0.23	5.6 ± 0.13	5.9 ± 0.11
**mocestinostat**	6.7 ± 0.13	6.7 ± 0.14	7 ± 0.18	<4	<4	<4	5.8 ± 0.02
**SAHA**	7.1 ± 0.02	6.9 ± 0.08	6.5 ± 0.23	6.7 ± 0.51	5.6 ± 0.15	6.4 ± 0.24	6.5 ± 0.001
**TSA**	7.1 ± 0.20	7 ± 0.16	6.6 ± 0.20	6.1 ± 0.16	5.5 ± 0.15	5.9 ± 0.06	6.9 ± 0.08

Supplementary Table 2. Target engagement and antiproliferative profiles of selected HDAC inhibitors in HeLa cells. Values are means of 3 independent experiments ± S.D.

Supplementary Methods

Tracer design & synthesis

Reagents were purchased from Sigma Aldrich and used without further purification unless otherwise noted. 4-(t-Butoxycarbonylaminoethyl)piperazine was purchased from Oakwood Chemical. The preparations of suberoyl[4-(aminomethyl)anilide] hydroxamic acid[1] and N-(5-aminopentyl)-2-[(4S)-6-(4-chlorophenyl)-1-methyl-8-(methyloxy)-4H-[1,2,4,]triazolo[4,3-a][1,4]benzodiazepine-4-yl]acetamide trifluoroacetate (iBET762 pentylamine) have been previously reported (Bailey, J.; Gosmini, R.; Luc, M.; Mirguet, O.; Witherington, J. WO 2011/054845 A1 2011). Silica gel chromatography was performed on a Teledyne Isco CombiFlash system. NMR spectra were acquired on a Varian Mercury 300 MHz system and referenced to internal solvent peaks. Reactions and products were analyzed on an Agilent 1100

Series analytical HPLC operated with EZChrome software, and preparative HPLC was performed on a Waters HPLC system with linear gradients as listed where A = 0.1% aqueous trifluoroacetic acid and B = acetonitrile, at a flow rate of 25 mL/min (1” C18 silica column). Low-resolution mass spectral data were acquired on a Waters 3100 Mass Detector optionally coupled with a Waters e2695 separations module and 2998 photodiode array detector and operated with MassLynx software v 4.1 (2010). HRMS data (ESI-TOF) were acquired by The Scripps Research Institute.

iBET-NCT

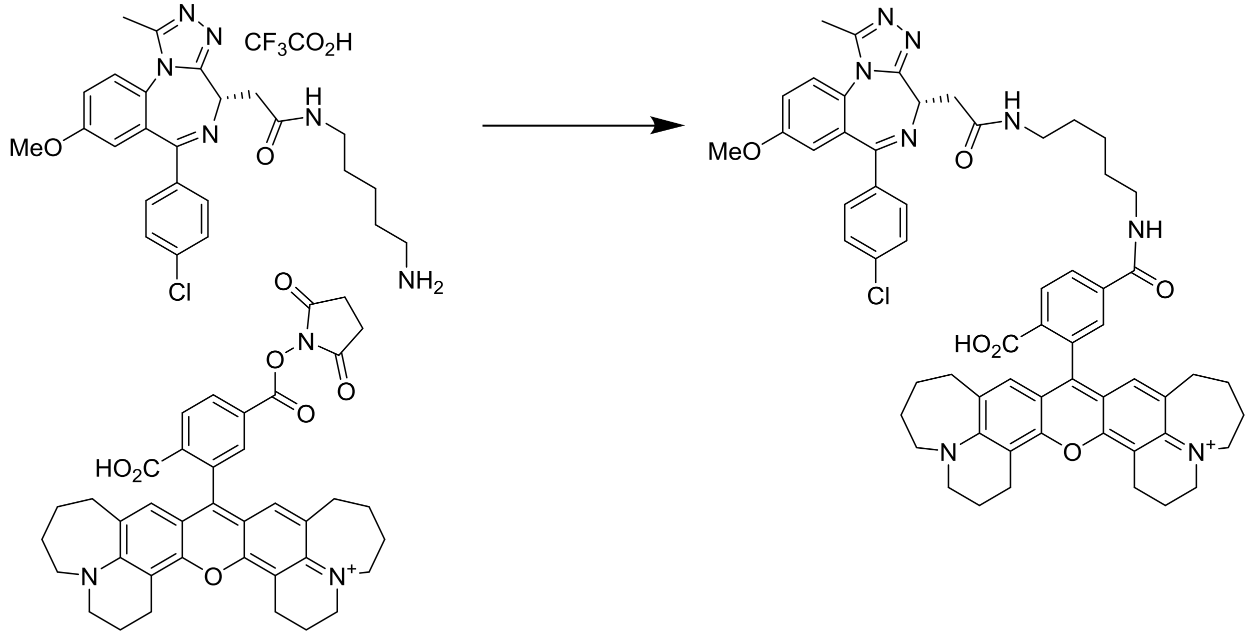

iBET-NCT was prepared as described in the literature using NCT-SE in place of AlexaFluor 488 (Bailey, J.; Gosmini, R.; Luc, M.; Mirguet, O.; Witherington, J. WO 2011/054845 A1 **2011)**. ^{1}H NMR (DMSO-d_6): δ 8.72 (br t, 1 H); 8.17-8.29 (m, 3 H); 7.77-7.80 (m, 2 H); 7.44-7.52 (m, 4 H); 7.38 (dd, 1 H); 6.86 (d, 1 H); 6.61 (s, 2 H); 4.44-4.49 (m, 1 H); 3.78 (s, 3 H); 3.64-3.72 (4 H); 3.49-3.55 (m, 4 H); 3.02-3.31 (m, 6 H);

2.93-2.99 (m, 4 H); 2.73-2.81 (m, 4 H); 1.94-2.02 (m, 4 H); 1.82-1.89 (m, 4 H); 1.68-1.76 (m, 4 H); 1.43-1.58 (m, 8 H); 1.31-1.39 (m, 2 H). HRMS: Calcd for $C_{60}H_{61}ClN_8O_6^+$: 1025.4475, found 1025.4480. Analytical HPLC: 96.9% @ 597 nm.

4968 SAHA-NCT

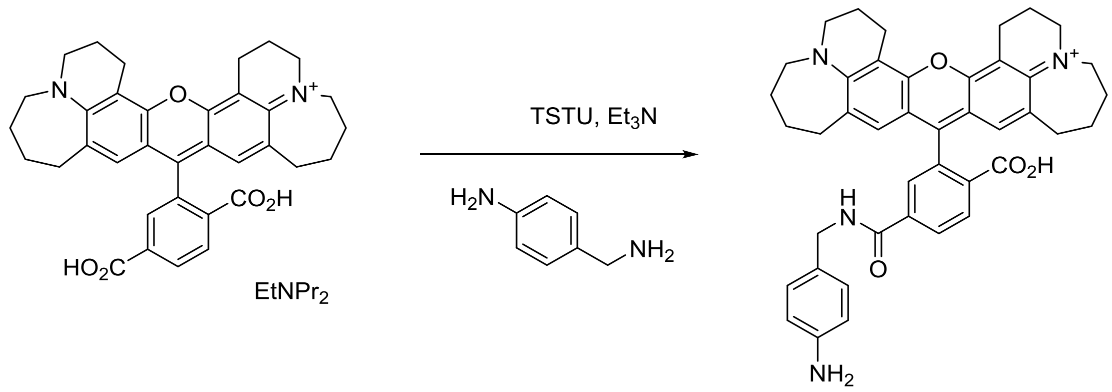

NCT acid (25 mg, 44 µmol) was stirred in 1 mL of DMF and treated with triethylamine (40 µL, 300 µmol) and TSTU (16 mg, 53 µmol). After 40 min, this reaction was added to a solution of 4-aminobenzylamine (54 mg, 442 µmol) in 0.5 mL of DMF and stirred for 1 h. The reaction was then acidified with AcOH, diluted with H2O and MeCN, and subjected to preparative HPLC (25->100% MeCN). The appropriate fractions were concentrated and lyophilized to afford 16 mg of a dark blue solid (54% yield). ^{1}H NMR (MeOH-d_4): δ 9.34 (br t, < 1 H); 8.38 (d, 1 H); 8.20 (dd, 1 H); 7.79 (d, 1 H); 7.52 (d, 2 H); 7.33 (d, 2 H); 6.67 (s, 2 H); 4.62 (s, 2 H); 3.72-3.76 (m, 4 H); 3.54-3.58 (M, 4 H); 3.02-3.08 (m, 4 H); 2.80-2.85 (m, 4 H); 2.65 (br t, < 1 H); 2.04-2.12 (m, 4 H); 1.92-2.01 (m, 4 H); 1.81-1.89 (m, 4 H). MS: Calcd for $C_{42}H_{43}N_4O_4^+$: 667.3; found 667.8. Analytical HPLC: 99.2% @ 600 nm.

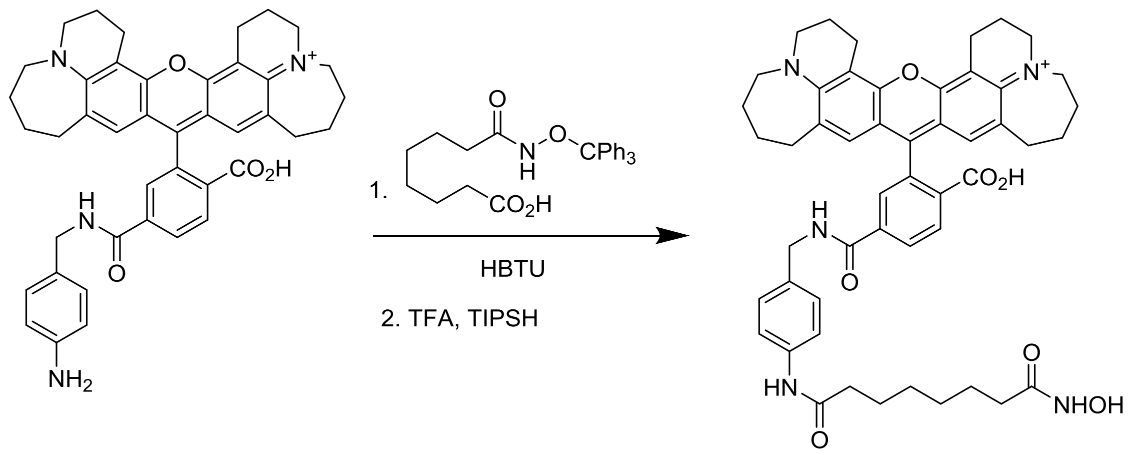

NCT aminobenzylamine adduct (16 mg, 24 µmol) was combined with O-trityl suberoylhydroxamic acid (20 mg, 46 µmol) and HBTU (26 mg, 70 µmol) in 1 mL of DMF and stirred overnight. The reaction was subjected to preparative HPLC (40->100% MeCN) and the appropriate fractions (MS Calculatedd for $C_{69}H_{70}N_5O_7^+$: 1080.5, found 1080.9. Analytical HPLC: 96% @600 nm) were combined, concentrated and lyophilized. The resulting dark blue solid (16 mg, 14.8 µmol) was dissolved in 2 mL of DCM and stirred in an ice bath. Triisopropyl silane (0.75 mL) and TFA (0.25 mL) were added and the reaction was monitored by HPLC. After 25 min the reaction was concentrated under reduced pressure. Some excess silane was removed by pipet; the remainder of the reaction was taken up in MeCN/H_2O and the product was isolated by preparative HPLC (25->100% MeCN in 0.1% aqueous TFA). Lyophilization of the appropriate fractions yielded 7.2 mg (58%) of the desired product. ^{1}H NMR (DMSO-d_6) δ 10.31 (br s, < 1H); 9.82 (s, 1 H); 9.23 (br t, < 1 H); 8.27 (s, 2 H), 7.81 (s, 1 H); 7.52 (d, 2 H); 7.24 (d, 2 H); 6.62 (s, 2 H); 4.38-4.45(m, 2 H); 3.66-3.73 (m, 4 H); 3.49-3.56 (m, 4 H); 2.91-2.99 (m, 4 H); 2.74-2.82 (m, 4 H); 2.26 (t, 2 H); 1.81-2.02 (m, 10 H); 1.70-1.78 (m, 4 H); 1.43-1.58 (m, 4 H); 1.21-1.31 (m, 4 H). HRMS: Calcd for $C_{50}H_{56}N_5O_7^+$: 838.4174; found 838.4172. Analytical HPLC: 94.2% @ 254 nm, 97.0% @600 nm.[1,2]

BIBF-NCT

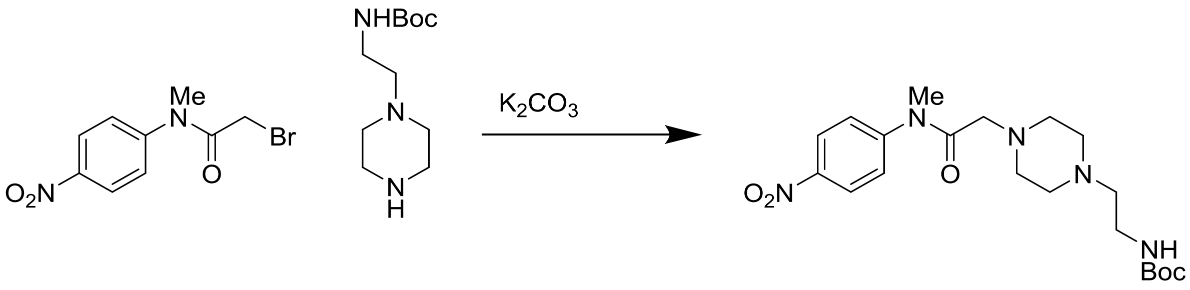

To a solution of 2-(Boc)aminoethylpiperazine (340mg, 1.5mmol) and K_2CO_3 (316 mg, 2.3 mmol) in acetone was added 400 mg (1.46 mmol) of 2-bromo-N-methyl-N-(4-nitrophenyl)acetamide. The reaction was stirred for 1.5 h at RT, then the solids were filtered off and the resulting solution was concentrated by rotary evaporation, taken up in EtOAc, and washed 2x with H_2O. The organic layer was dried over Na_2SO_4 and evaporated to afford 274 mg (44% yield) of a slightly yellow solid residue. ^{1}H NMR ($CDCl_3$) 8.27 (d, 2 H); 7.43 (d, 2 H); 5.00 (br tr, < 1 H); 3.35 (s, 3 H); 3.17-3.26 (m, 2 H); 3.06 (s, 2 H); 2.39-2.55 (m, 10 H); 1.43 (s, 9 H). ^{13}C NMR ($CDCl_3$) 169.12, 155.95, 149.46, 146.26, 127.25, 124.84, 79.28, 60.34, 57.11, 52.70 (2), 37.45, 37.00, 28.44. MS: Calcd for $C_{20}H_{32}N_5O_5^+$: 422.2, found 422.2. A small amount of the material was further purified by preparative HPLC and isolated as the TFA salt. ^{1}H NMR (MeOH-d_4) δ 8.30-8.36 (m, 2 H); 7.58-7.64 (m, 2 H); 3.34 (m, 2 H); 3.36 (s, 3 H); 3.19-3.27 (m, 4 H); 3.08 (t, 2 H); 2.91-2.99 (m, 4 H); 1.44 (s, 9 H). ^{13}C NMR (MeOH-d_4) δ 168.83, 158.81, 149.56, 148.38, 129.27, 126.19, 81.09, 59.09, 57.87, 52.36, 51.21, 37.84, 36.63, 28.78.

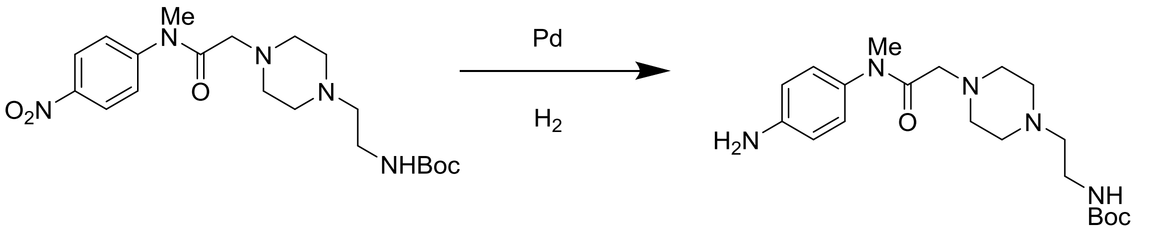

To a flask equipped with a stir bar was added Pd/C (10 mol%, 32mg, 30µmol). The flask was purged with nitrogen gas, and a solution of 140 mg (0.332 mmol) of N-methyl-2-(4-aminoethylpiperazin-1-yl)-N-(4-nitrophenyl)acetamide in degassed methanol was added. The reaction was stirred under an H_2 atmosphere overnight, then filtered through Celite and evaporated to yield the desired product (137 mg, 105% yield). MS: Calcd for $C_{20}H_{34}N_5O_3^+$: 392.3, found 392.2. The crude material was used directly in the next reaction, although a small amount was purified by preparative HPLC (2->50% B) for characterization as the TFA salt: ^{1}H NMR (MeOH-d_4) δ 7.38-7.47 (m, 4 H); 3.63 (s, 2 H); 3.37-3.52 (m, 8 H); 3.28 (s, 3 H); 3.15-3.23 (m, 4 H); 1.43 (s, 9 H).

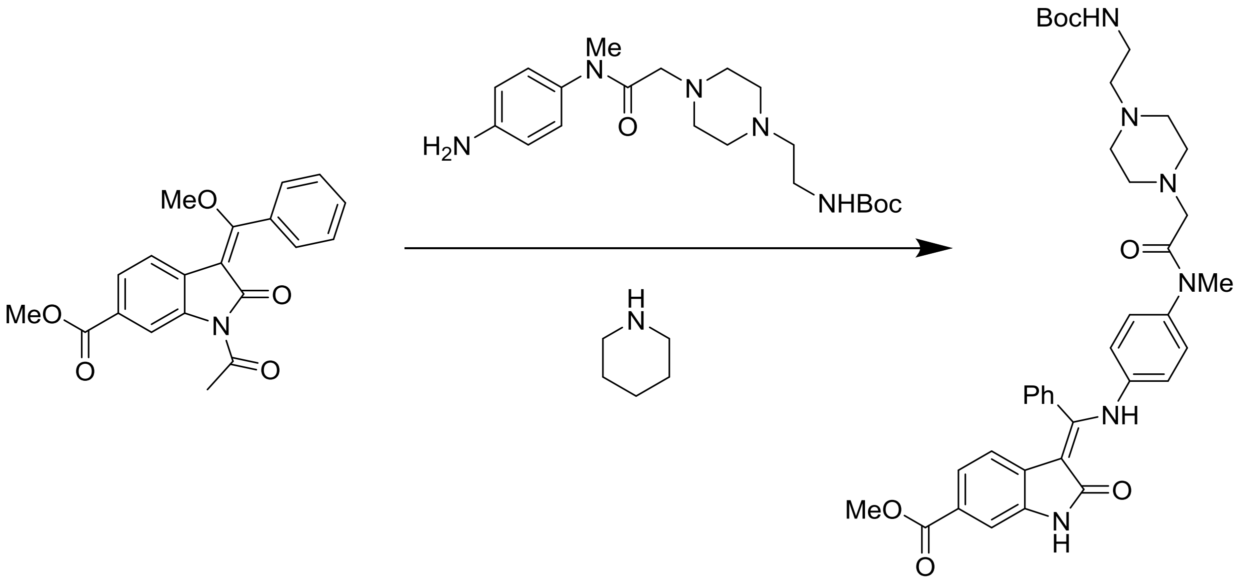

6-Acetyl-2-oxo-2,3-dihydro-1H-indole-6-carboxylic acid methyl ester (110 mg, 0.313 mmol) and N-methyl-2-(4-aminoethylpiperazin-1-yl)-N-(4-aminophenyl)acetamide (121 mg, 0.310 mmol) were combined in 1mL of DMF and heated to 80 C for 30 min. The reaction was then cooled to RT, and 60 µL (0.282 mmol) of piperidine was added. After 40 min the reaction was quenched by addition of water, diluted with brine, and extracted with 3 x EtOAc. The combined organic layers were dried with Na_2SO_4 and concentrated by rotary evaporation. The product was obtained by column chromatography eluting with a gradient from 0->20% MeOH in DCM as 80 mg (42% yield) of a yellow-brown solid. ^{1}H NMR

(CD_2Cl_2): δ 7.94 (br s, 1 H); 7.51-7.62 (m, 4 H); 7.42-7.46 (m, 2 H); 7.33 (dd, 1 H); 6.96-7.04 (m 2 H); 6.80-6.87 (m, 2 H); 6.01 (d, 1 H); 4.96 (br s, 1 H); 3.82 (s, 3 H); 3.42 (s, 5 H); 3.10-3.19 (m, 4 H); 2.30-2.45 (m, 8 H); 1.42 (s, 9 H). ^{13}C NMR ($CDCl_3$) δ 170.947, 169.49, 167.48, 158.50, 156.18, 140.05, 138.25, 135.48, 132.52, 130.83, 129.87, 129.30, 128.75, 128.03, 125.46, 124.10, 123.09, 118.49, 110.42, 98.56, 79.48, 59.55, 57.33, 52.95(2), 52.14, 37.55, 37.09, 28.64. MS: Calcd for $C_{37}H_{45}N_6O_6^+$: 669.3; found 669.7.

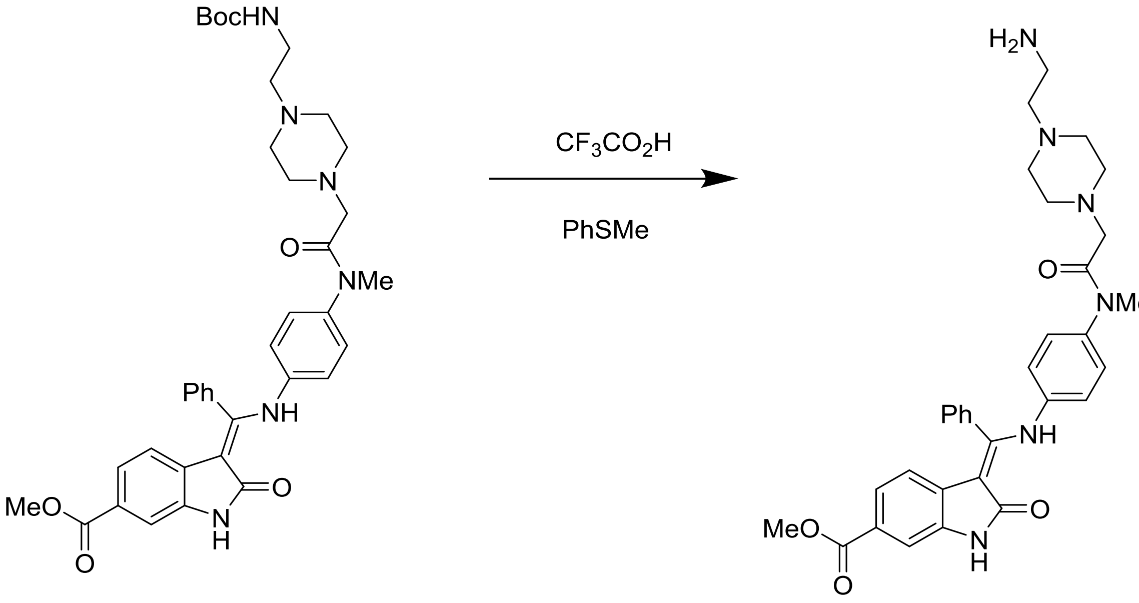

Z)-3-({4-[2-(4-(2-(N-Boc)aminoethyl)piperazin-1-yl)acetylamino]-phenylamino}-phenyl-methylene)-2-oxo-2,3-dihydro-1H-indole-6-carboxylic acid methyl ester (15.9 mg, 24 µmol) was dissolved in 0.25 mL of DCM and stirred in an ice bath. Thioanisole (25 µL) and TFA (0.25 mL) were added and the reaction was monitored by HPLC. After 30 min, the reaction was concentrated under reduced pressure, dried under vacuum, and then taken up in H_2O. The product (15 mg, 92%) was isolated via preparative HPLC eluting with 5->100% MeCN in 0.1% aqueous TFA. ^{1}H NMR (MeOH-d_4) δ 7.58-7.68 (m, 5 H); 7.54 (d, 1 H); 7.49 (dd, 2 H); 7.27 (dd, 1 H); 7.11-7.19 (m, 2 H); 6.87-6.97 (m, 2 H); 5.94 (d, 1 H); 3.83 (s, 3 H); 3.78 (s, 2 H); 3.22 (s, 3 H); 3.27 (br s, 2 H); 3.05-3.08 (m, 2 H); 2.81 (br s, 6 H); 2.69-2.73 (m, 2 H). ^{13}C NMR (MeOH-d_4): δ 172.53, 169.08, 164.82, 159.82, 140.73, 138.58, 137.87,

133.93, 132.03, 131.10, 130.55, 129.96, 129.35, 126.60, 125.29, 124.56, 119.50, 111.45, 100.23, 57.89, 54.77, 54.05, 52.60, 50.51, 37.93, 37.40. MS: Calcd for $C_{32}H_{37}N_6O_4^+$: 569.3, found 569.6.

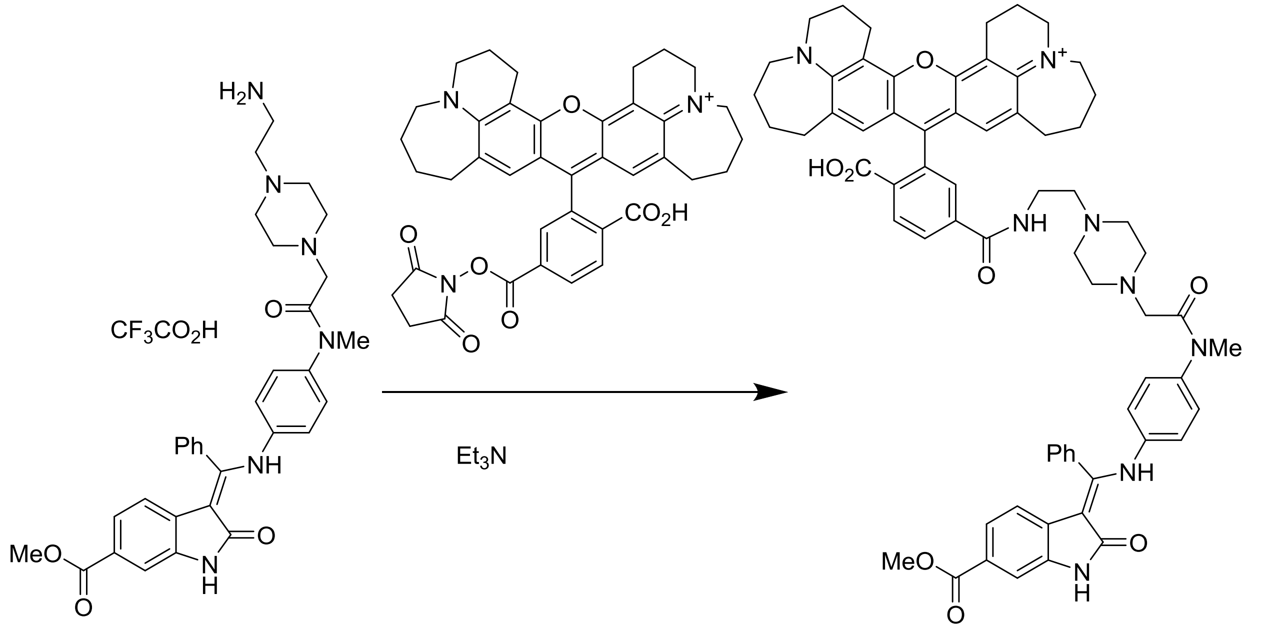

Z)-3-({4-[2-(4-(2-aminoethyl)piperazin-1-yl)acetylamino]-phenylamino}-phenyl-methylene)-2-oxo-2,3-dihydro-1H-indole-6-carboxylic acid methyl ester trifluoacetate salt (6 mg, 8.8 µmol) was combined with 5.2 mg (7.9 µmol) of NCT-SE and 5 uL of triethylamine in 1 mL of DMF. After 30 min, the reaction was acidified with AcOH, diluted with MeCN and H_2O, and the desired product was isolated by preparative HPLC eluting with 25->100% MeCN in 0.1% aq TFA. After concentration and lyophilization 6 mg (68% yield) of a purple solid was obtained. ^{1}H NMR (DMSO-d_6) δ 12.27 (s, 1 H); 10.98 (s, 1 H); 8.88 (br s, 1 H); 8.29 (d, 1 H); 8.22 (dd, 1 H); 7.77 (s, 1 H); 7.52-7.66 (m, 7 H); 7.43 (s, 1 H); 7.15-7.21 (m, 4 H), 6.89 (d, 2 H); 6.59 (s, 2 H); 5.84 (d, 1 H); MS: Calcd for $C_{67}H_{68}N_8O_8^+$: 1113.5233, found 1113.5234. Analytical HPLC: 97.7% @ 254 nm, 98.7% @ 600 nm.[3-6]

Supplementary References

1 Schafer, S. *et al.* Phenylalanine-containing hydroxamic acids as selective inhibitors of class IIb histone deacetylases (HDACs). *Bioorganic & medicinal chemistry* **16**, 2011-2033, doi:10.1016/j.bmc.2007.10.092 (2008).

2 Salisbury, C. M. & Cravatt, B. F. Activity-based probes for proteomic profiling of histone deacetylase complexes. *Proceedings of the National Academy of Sciences of the United States of America* **104**, 1171-1176, doi:10.1073/pnas.0608659104 (2007).

3 Hilberg, F. *et al.* BIBF 1120: triple angiokinase inhibitor with sustained receptor blockade and good antitumor efficacy. *Cancer research* **68**, 4774-4782, doi:10.1158/0008-5472.CAN-07-6307 (2008).

4 Roth, G. J. *et al.* Design, synthesis, and evaluation of indolinones as triple angiokinase inhibitors and the discovery of a highly specific 6-methoxycarbonyl-substituted indolinone (BIBF 1120). *Journal of medicinal chemistry* **52**, 4466-4480, doi:10.1021/jm900431g (2009).

5 Roth, G. J. *et al.* Design, synthesis, and evaluation of indolinones as inhibitors of the transforming growth factor beta receptor I (TGFbetaRI). *Journal of medicinal chemistry* **53**, 7287-7295, doi:10.1021/jm100812a (2010).

6 Ku, X., Heinzlmeir, S., Helm, D., Medard, G. & Kuster, B. New affinity probe targeting VEGF receptors for kinase inhibitor selectivity profiling by chemical proteomics. *Journal of proteome research* **13**, 2445-2452, doi:10.1021/pr401247t (2014).
